# Lifestyle Medicine Pillars and Pedagogies in Pre-registration Health Profession Degrees: A Scoping Review

**DOI:** 10.1007/s40670-025-02359-y

**Published:** 2025-03-17

**Authors:** Jack Natin, Muhammad Ahmad Ashfaque, Anne Hickey, Frank Doyle, Maria Pertl

**Affiliations:** School of Population Health, RCSI University of Medicine and Health Sciences, Dublin, Ireland

**Keywords:** Lifestyle medicine, Education, Non-communicable disease, Healthcare students, Preventative medicine, Teaching methodologies

## Abstract

Non-communicable diseases are the greatest cause of mortality and disability globally. Lifestyle medicine (LM) can facilitate effective prevention and treatment of such diseases. However, the extent and nature of LM education has not been sufficiently mapped. This scoping review aimed to assess the pedagogies, content and structure of LM education in pre-registration health profession degrees using the JBI methodology. Fifty-two articles met the inclusion criteria. Three of the six core LM pillars were not taught in the majority of programmes studied. Universities should survey their curricula to identify such gaps and appropriate opportunities to incorporate these pillars into existing modules.

**Supplementary Information:**

The online version contains supplementary material available at 10.1007/s40670-025-02359-y.

## Introduction

Non-communicable diseases have become the leading cause of global death in recent decades. The World Health Organisation estimates that 74% of all deaths in 2019 occurred because of such chronic diseases [1]. It is now widely recognised that the most effective way of mitigating these diseases is with lifestyle modifications [1–4]. Lifestyle medicine (LM) has emerged in recent years as a holistic approach for the prevention and treatment of non-communicable disease. It is a type of healthcare intervention delivered by clinicians that uses therapeutic lifestyle interventions as the basis of treatment for chronic diseases [5]. The potential of LM as a preventative measure — as well as an effective treatment — makes it particularly useful as a means of reducing both the financial and mortality burdens of non-communicable disease. The global financial burden of physical inactivity alone was estimated to be $ (Int) 67.5 billion annually [6], while inadequate quality sleep is estimated to cost the United States $ (USD) 14 billion each year [7]. Effective LM delivery has the potential to reduce these costs as well as improving other factors such as worker productivity [7] and disability-adjusted life years [8]. In order to reap these potential benefits, we need to adequately train healthcare professionals to deliver LM interventions.

LM revolves around six pillars of positive health: nutrition, physical activity, avoidance of risky substances, restorative sleep, stress management and social connections [5, 9]. The European Lifestyle Medicine Organisation expands this to include two additional pillars of environmental exposure and sexual health [10]. These pillars of LM have developed from principles of healthy lifestyle and health behaviour change that have existed for millennia, for example, Hippocrates stated that ‘in order to keep well, one should simply avoid too much food, too little toil’ [11]. More recent evidence-based lifestyle interventions such as motivational interviewing and smoking cessation are also well-established and broadly employed [12, 13]. LM as an entity distinct from these prior developments emerged primarily due to an acknowledgment that physicians were not adequately employing many of these evidence-backed interventions. For example, despite increasing evidence that exercise is one of the most effective interventions for improving general health [14], a study of US physicians in 2012 found that only 30% of those surveyed had provided exercise counselling in the 12 months prior [15]. Healthcare professionals have expressed that insufficient knowledge remains a significant barrier to following recommendations on implementing such interventions [16–19]. LM provides a framework for ensuring healthcare professionals receive necessary foundations in key domains of healthy lifestyle so that they can support patients to make positive lifestyle changes that can impact their health. It allows for a clear re-orientation of global healthcare towards preventative measures and because it is an evidence-based field, it also allows for better standardisation of lifestyle interventions.

The first LM institution, the American College of Lifestyle Medicine, was established in 2004 [5]. Several international institutions were founded even more recently: the Lifestyle Medicine Global Alliance was initiated in 2015 by the American College of Lifestyle Medicine [20], and the International Board of Lifestyle Medicine was founded later in 2017 [9]. While this suggests progress towards LM integration into healthcare practices, recent research suggests that lifestyle interventions are still not sufficiently employed by healthcare professionals [21–23]. To improve the ability of clinicians to provide lifestyle counselling, it is necessary to provide them with adequate training. Placing such instruction in pre-registration healthcare degrees would ensure all healthcare professionals received adequate LM education. This review therefore aims to specifically scope the extent and nature of LM as an emerging aspect of healthcare education.

The inclusion of LM in medical education has implications for the teaching methodologies used in educational activities. LM places increased emphasis on the role of healthcare practitioners as health counselors or coaches [24, 25]. While all healthcare professionals must motivate their patients to adhere to treatments, with LM, the requirement for this skill is even more pronounced, as the treatments are constant and perpetual — by definition, lifestyle modifications are changing the way patients live their lives. To prepare healthcare professionals to deliver such increased levels of motivational and behavioural support, an increased focus on interpersonal skills and techniques, such as motivational interviewing and behaviour change theories, is required [25]. It has been suggested that active or experiential learning methodologies are appropriate for teaching these skills [26–28]. This review investigates the variety of teaching methodologies that are currently being employed in LM education. The review also investigates how these teaching methodologies are being evaluated. As an evidence-based field of practice, it is important that the education of LM is as thoroughly tested as LM interventions themselves.

The review builds on evidence from an existing review which documented the teaching of each pillar of LM and how these might be integrated into occupational therapy [29]. Our review differs from the pre-existing review by using a broader search strategy and documenting a wider range of program characteristics.

The aim of this review is to examine how LM is included in the pre-registration degrees of healthcare professionals. Within this research question, our objectives are (1) to assess the extent to which each of the pillars of LM are covered, (2) to identify the prominent pedagogical approaches which are employed to teach LM and (3) to describe how research is conducted to evaluate the teaching of LM. It is hoped that by detailing the extent, location and nature of current teaching and of available literature in this area, recommendations for implementing LM into basic healthcare training can be made.

## Materials and Methods

The review was developed in line with the JBI guidelines for scoping reviews [30], and reported according to the Preferred Reporting Items for Systematic Reviews and Meta-Analyses extension for Scoping Reviews (PRISMA-ScR) checklist (see online resource A: Appendix I) [31]. This review was conducted in accordance with an a priori protocol, which was published in the Open Science Framework [32]. Any deviations from this protocol are reported and justified in Appendix II (see online resource A: Appendix II).

### Inclusion Criteria

The inclusion criteria are detailed and justified in the a priori protocol relating to this review [32]. An overview is provided here.

#### Participants

The review is specifically concerned with the teaching of LM to healthcare professional students. Participants are limited to students of the following common healthcare professions: nurses, doctors/physicians, physician associates, psychologists, pharmacists, physiotherapists, dieticians, midwives, dentists, podiatrists, social workers or occupational therapists.

#### Concept

The review is limited to literature which examines the teaching of lifestyle medicine, whereby only literature that explicitly mentions the term ‘Lifestyle Medicine’ will be included. This term relates to a specific structured approach to healthcare intervention that has developed around eight core pillars: physical activity, nutrition, sleep, avoidance of risky substances, social connection and stress reduction, environmental exposure and sexual health. It is, therefore, distinct from more general discussions about preventative medicine or lifestyle interventions, as described previously.

#### Context

The review includes literature which pertains to pre-registration healthcare profession students. Literature pertaining to post-qualification LM education is excluded because the paper aims to map the integration of LM prior to registration, rather than the availability of post-graduate qualifications of which many healthcare professionals may not avail.

#### Types of Sources

This scoping review considers peer-reviewed qualitative, quantitative and mixed methods studies. Systematic reviews, scoping reviews, case reports and conference abstracts have also been included. In addition, a search of grey literature was undertaken.

### Exclusion Criteria

1. Literature that does not pertain to the following healthcare professions: nurses, doctors/physicians, physician associates, psychologists, pharmacists, physiotherapists, dieticians, midwives, dentists, podiatrists, social workers or occupational therapists.
2. Literature that does not pertain to pre-registration degrees of the above healthcare professions.
3. Literature that does not specifically mention the term ‘Lifestyle Medicine’.
4. Literature that is not published in English was excluded due to lack of translational resources available.

### Search Strategy

The search strategy aimed to locate both published and unpublished studies in a four-stage process. Prior to beginning, in collaboration with an information specialist librarian, a pilot search of Ovid MEDLINE, Embase, EBSCO CINAHL, Scopus and Web of Science was performed to identify keywords relating to the population, concept and context being examined. The research team then compiled a list of relevant terms and synonyms to be used in the search.

The first stage of the strategy was a search of the above databases using the finalised search strategy (an example of the search strategy for Ovid MEDLINE is included in online resource A: Appendix III), conducted on 28 June 2024. In the second stage, a manual review of the reference lists and citations of included literature was conducted by the screeners to search for additional relevant literature. Articles that cite the included articles were then reviewed for inclusion via the Google Scholar ‘cited by’ function. Finally, a targeted search of the sister organisations of the Lifestyle Medicine Global Alliance was also undertaken. Any relevant documents linked to eligible sources (e.g. conference abstracts) identified in the searches were also included.

### Study/Source of Evidence Selection

**Fig. 1:**
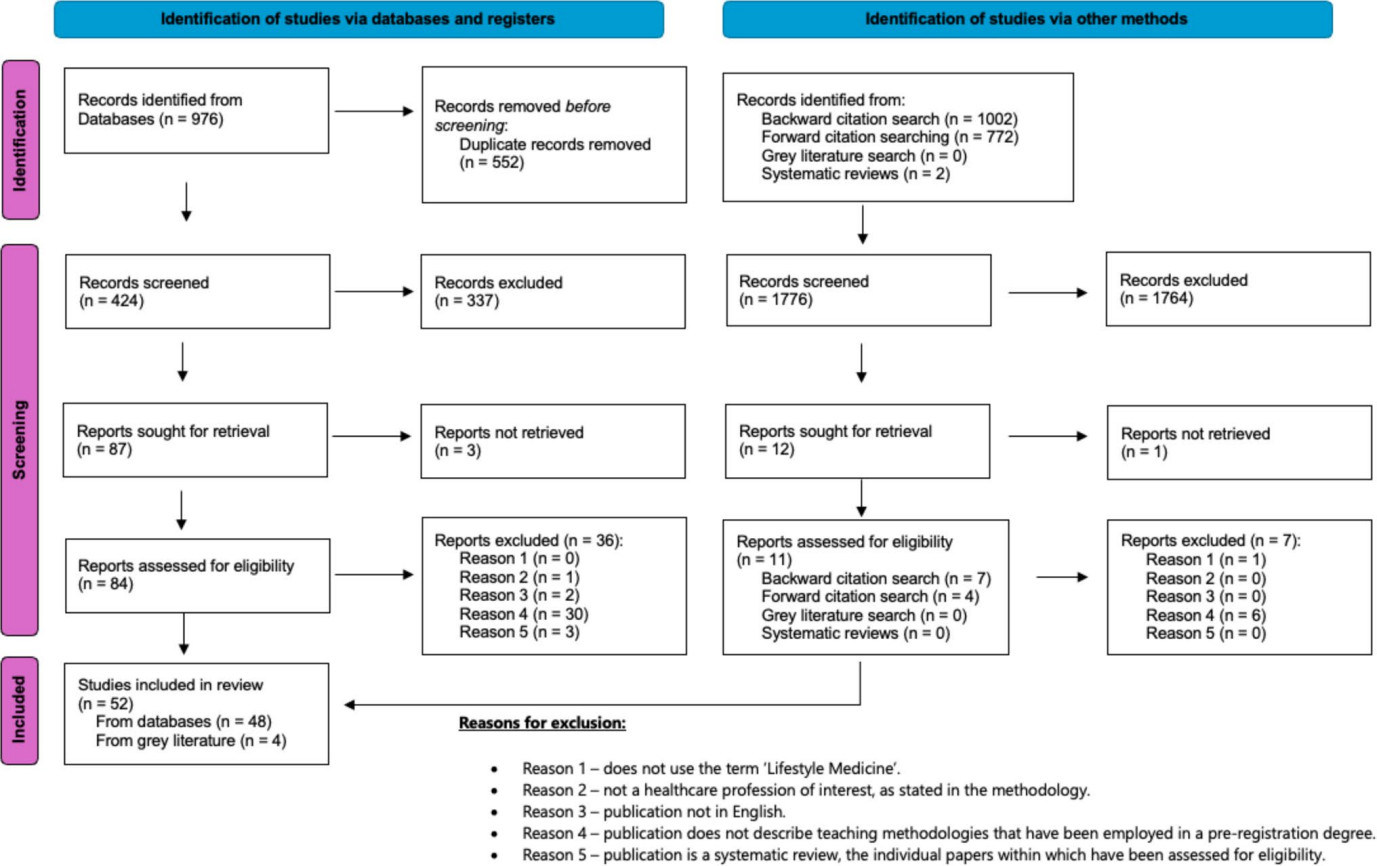
Modified PRISMA 2020 flow diagram of search and screening results

All database search results were collated and imported into EndNote 21. Duplicate records were removed. Following a pilot test, titles and abstracts were then screened by two independent reviewers for assessment against the inclusion/exclusion criteria for the review. Literature selected for full-text screening were downloaded and the full-text articles were reviewed against the inclusion/exclusion criteria. The reasons for the exclusion of any full-text article are presented in a PRISMA flow diagram in Fig. 1 [33]. Disagreements between screeners were resolved by discussion.

### Data Extraction

Data was extracted from papers included in the scoping review by two independent reviewers. The data extracted included specific details about the participants, concept, context and key findings relevant to the review questions. The following entities were extracted from each article: author/s, year of publication, country, degree taught, year of degree taught, pillars of LM taught, tutor’s profession, elective or mandatory nature of programme, accreditations awarded for programme, teaching methodologies employed, synchronicity of programme, mode of content delivery (online/in-person/hybrid), frequencies of classes, hours of tutelage and method of programme evaluation (including student knowledge and skills assessments).

The draft extraction tool for these entities was tested on the first five papers. It was modified to include the entity ‘university’ as many research papers detailed new programmes in the same university. Disagreements that arose between the reviewers were resolved through discussion. Authors of papers were contacted to request access to papers when necessary or to request additional information if teaching methodologies were absent from the paper.

### Data Analysis and Presentation

The entities extracted from the review are descriptively detailed in Table 1 (see online resource B). The teaching methodologies, frequency of classes and programme assessment methodologies were coded into categories in Table 2 (see online resource C) to aid data presentation. For example, methodologies such as clinical placements and physician shadowing were coded into the ‘clinical shadowing’ category, and programme evaluations such as BMI measurements and GAD-7 scores were coded into the ‘quantitative assessments’ category. For the frequency of classes, programmes which had gaps of at least a day between several LM teaching activities were coded into ‘disseminated’ learning, while programmes that had all LM teaching activities occurring on successive days were coded into ‘block delivery’.

## Results

The review found 52 research articles which met eligibility criteria. These 52 articles had details of 71 teaching programmes. This included programmes in 37 different universities, as well as 7 programmes which taught pre-registration healthcare students but either did not specify the university or were taught by an organisation that was not a university — for example, the VA Boston Healthcare System. The full results of data extraction are detailed for each programme in Table 1 (see online resource B) [2, 34–84], and the key findings across all programmes have been compiled and presented in Table 2 (see online resource C). Table 3 (see online resource D) collates and presents the results by university rather than by programme, to allow easier comparison between institutions. The majority of universities were in the USA (*n* = 30), with others in Germany (*n* = 3), Israel (*n* = 2), the UK (*n* = 1) and Pakistan (*n* = 1). Nearly all programmes (of the 68 which specified) (87%) were taught to medical students, 9% taught to physician associates, with pharmacy, nursing and ‘all allied health professionals’ taught by one programme each. Teaching was slightly more likely to occur in earlier years. Within the 55 programmes which specified the year being taught, 69% and 67% of programmes taught first- and second-year students, respectively, compared to 56% and 44% respectively for third- and fourth-year students.

**Fig. 2:**
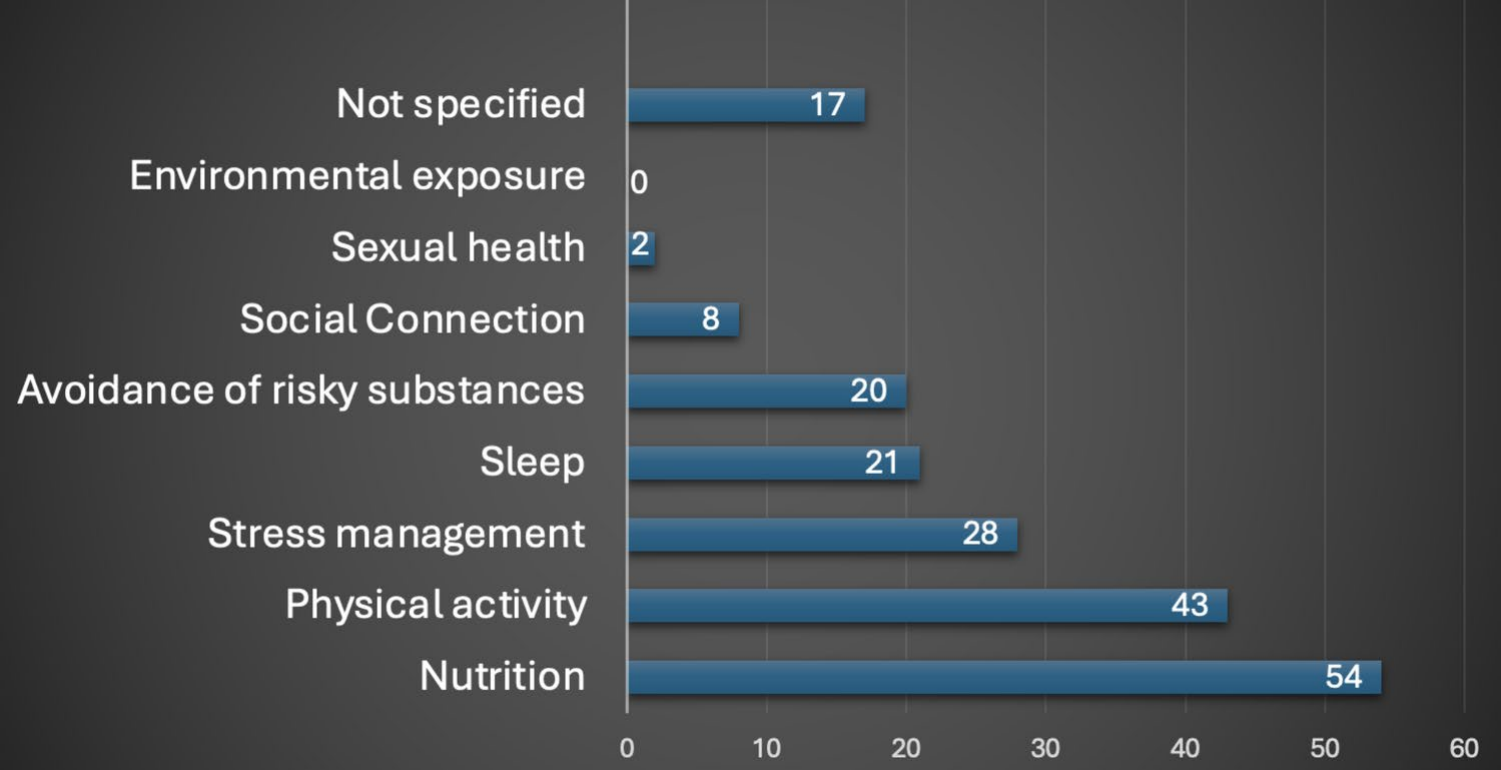
The number of programmes teaching each LM pillar

The most commonly taught LM pillars are shown in Fig. 2. Within the 54 programmes which specified the pillars of LM being taught, nutrition and physical activity were by far the most commonly taught pillars, with all of these programmes teaching nutrition and 80% teaching physical activity. Just over half of the programmes taught stress management (52%), while sleep and avoidance of risky substances were less common, featuring in 39% and 37% of programmes respectively. Despite being a core pillar of LM [5, 9], social connection was poorly represented in teaching — with only seven programmes in six universities teaching it. The additional two pillars — sexual and environmental health — which the European Lifestyle Medicine Organisation endorses [10] were nearly entirely absent. Sexual health was only included in two programmes. Environmental health was not formally taught in any programme.

Tutors from a wide variety of professions taught LM programmes, with 34 programmes specifying the tutor’s profession. In these programmes, the tutors were most commonly physicians (44% of programmes), dieticians (38% of programmes) and chefs (35% of programmes). Of the 56 programmes which specified, most were elective (46%); however, there were a significant number that were either mandatory (34%) or consisted of both mandatory and elective components (20%). The vast majority of programmes did not specify an award for completion of the LM programme (73%). Of those that did, credit (42%) or incorporation into the main degree grades (42%) were the most common awards. There were also two programmes that specified that no award was given for programme completion [50], one programme which offered optional culinary medicine certification [53] and one programme entered students into a raffle to win a $50 gift card [57].

Teaching methodologies were specified by 66 programmes and a wide variety were employed — 62% of these programmes using a methodology to teach LM that was either unique to that programme or employed by no more than one other programme (listed as ‘other’ in Table 2 — see online resource C). Examples of these unique methodologies included field trips [34], virtual grocery store tours [43], coaching elementary school students [45], preparing plant-based lunches [36] and more. The most common teaching methodologies were lectures (62%), cooking activities (35%), group discussions (33%), case studies (27%) and patient counselling (26%). Also displayed in Table 2 (see online resource C) are 19 additional teaching methodologies that were included in 3 or more LM programmes.

The majority of programmes did not specify the synchronicity of the LM education (75%). The number of universities and programmes that were specified as synchronous, asynchronous or hybrid are listed in Table 2 (see online resource C). Notably, in 2019, the University of Central Florida offered a programme that was available either synchronously or asynchronously, but not as a hybrid [54]. Similarly, the majority of programmes did not specify whether classes were held online, in-person or in a hybrid fashion (63%), though the data for those that did is also presented in Table 2 (see online resource C).

As seen in Table 2 (see online resource C), most programmes were delivered in many classes disseminated across the academic year rather than in a single block of dedicated LM teaching. The three German universities offered either format to students [39]. The mean number of hours taught in the 39 programmes which specified their duration was 40 h, while the median value was 20 h. There were significant outliers, such as one programme which had 300 h of content [53] (the next highest was 86.5 [82]) and another programme which had only 20 min of content (the next lowest was 1 h [50]).

Details of programme evaluation were given by 40 programmes. Within these, Likert-scales or rating systems for criteria such self-rated understanding, confidence and knowledge were by far the most common measurement, used by 75% of programmes. Qualitative measurements such as open-ended questions were used by 58% of programmes. Many programmes (53%) asked students to complete quantitative measurements relating to student knowledge — such as MCQs — or relating to measurements lifestyle change — such as MedDiet scores and BMI values. Summative assessments of knowledge or competencies were used by 42% of programmes while the remaining 58% did not objectively measure such attainment, with many using student self-rated perceptions of knowledge acquisition instead.

## Discussion

This review is the first to synthesise both the content and pedagogical approaches to LM teaching and assessment for pre-registration healthcare professions internationally. The main findings are heterogeneity of content and approaches, including assessments, a varying elective-mandatory nature of programmes, the US-centric nature of published literature in this area, the lack of documented LM teaching in pre-registration healthcare degree courses and the frequent mismatch between programme objectives and programme assessments.

### Programme Content

The results of this review reveal a large disconnect between what international organisations promote as the core tenets of LM, and what is being taught in universities. The results suggest that there is significantly less importance placed on some pillars by educational institutions — most notably social connection, which is only taught in 15% of programmes studied. While international organisations recognise the importance of this pillar for contributing to mental health and longevity, this is not reflected in teaching practices [85]. This seems to be an oversight, given the increasing evidence associating poor physical and mental health outcomes with a lack of social connection [86, 87]. Excluding this pillar from LM education may leave future clinicians unprepared to deliver lifestyle interventions for mental health. This may result in lost opportunities to support patient health, and have health and economic implications by contributing to higher disease morbidity and a greater focus on more costly specialised care, such as counselling and medications, rather than preventative approaches and strategic use of community resources. For example, social prescribing has become a more prominent LM intervention in healthcare and represents a key opportunity to incorporate teaching on practical interventions for supporting lifestyle changes relating to this pillar [88, 89].

Avoidance of risky substances was only taught by 37% of programmes; however, it is likely that this particular pillar is taught in universities under a different guise than LM, given the widespread acknowledgment of the dangers of smoking and alcohol consumption. For example, ‘Making Every Contact Count’ is a lifestyle intervention widely employed and taught in the UK and Ireland to encourage all healthcare providers to discuss use of risky substances with patients [84, 90]. Such existing LM interventions should be surveyed to identify gaps and potential overlaps within curricula.

Sleep was taught by 39% of programmes. Unlike smoking and alcohol, it is less likely that sleep is taught in detail in healthcare education outside of LM modules. A 2011 study of 106 medical schools found that the mean number of hours spent on sleep education was less than 2.5 h and that 27% of programmes did not dedicate any time to sleep education [91]. Yet sleep remains one of the foundations of all aspects of health [92]. Barriers to the implementation of sleep education in healthcare programmes identified include a lack of time, insufficient qualified staff and the perception that it is a low priority [93], and it is likely these barriers apply to its inclusion in LM education as well. The holistic nature of LM represents a unique opportunity to link sleep interventions to a variety of different conditions, improving recognition of its importance in health whilst also providing practical interventions for healthcare professionals and their patients. For example, there are clear associations between obstructive sleep apnoea and cardiovascular health risks [94]. Lifestyle sleep interventions such as sleeping on one’s side can improve airflow for many patients with obstructive sleep apnoea, improving their cardiovascular health [95]. Sleep hygiene interventions also have been shown to have a variety of applications such as reducing rates of depression in patients dealing with substance abuse [96] and improving the quality of life for patients on dialysis [97]. LM education on sleep could therefore by integrated into a variety of modules such as the cardiovascular system, psychiatry or renal health. Incorporating LM interventions into existing modules may also reinforce the idea that LM should be a basic tenet of medical practice, rather than a possible addition for students with a particular interest in LM.

Stress management is a crucial element of LM, not only because it directly impacts patient well-being [98], but because it also influences a patient’s ability to engage with the other 5 core pillars of LM [99, 100]. The review finding that only 52% of programmes teach this pillar further evidences the gaps that remain in LM education. Opportunities to incorporate teaching on stress management in healthcare degrees are abundant. As with avoidance of risky substances, it is likely that stress management is already taught in some form in healthcare education, and it would be useful to survey curricula to identify if and where this occurs. Like all of the pillars of LM, there is the potential to teach stress management in almost any topic. Stress has important impacts on mental health [101], cardiovascular outcomes [102], child and adolescent development [103]. There are also less frequently discussed associations such as an increased risk of respiratory infections [104]. The possibility to introduce stress management techniques such as meditation, breathing exercises or journalling is present within all of these topics. Many universities are already teaching stress management directly to their students with the intention of helping them manage the pressures of their healthcare degrees [105]. Linking this type of intervention to the employment of LM with patients represents a particularly useful way of integrating this pillar into education programmes.

Physical activity was taught by 80% of programmes which specified their programme content, which likely reflects the well-established link between exercise and health outcomes. Its absence from 20% of programmes may seem peculiar; however, of the 11 programmes that did not include physical activity, 10 taught only nutrition (the remaining programme in the University of North Carolina taught Nutrition and Sexual Health only) [75]. This may indicate that physical activity was not omitted due to a disregard for its importance but possibly due to a restriction on the number of LM modules that could be implemented. It is unclear why nutrition is better represented than physical activity, though this does reflect wider trends in US medical schools. While 29% of medical schools in the USA provide 25 h of nutrition instruction, less than 20% offer courses relating to physical activity [53, 106, 107]. LM represents an opportunity to address these educational gaps. In particular, the topic of type 2 diabetes mellitus (T2DM) represents an appropriate area to integrate both physical activity and nutrition LM interventions. In 2022, 14% of people globally aged 18 or over were living with diabetes, with 95% of these people having T2DM [108]. The prevalence of the disease combined with the vast number of associated complications [109] make it likely that diabetes is taught by most pre-registration healthcare degrees. Research has found that lifestyle interventions are effective not only for preventing T2DM development [110], but for inducing remission and improving quality of life for those who have it [111]. Most of these interventions revolve around the pillars of physical activity and nutrition. Cooking activities were undertaken by 35% of programmes in this review and such an activity might be placed within endocrine modules where students will learn to counsel patients on how best to manage their diabetes. Lifestyle prescriptions, taught by 21% of programmes in the review, could be introduced to this topic in the form of dietary or exercise prescriptions.

Interestingly, a considerable number of programmes had listed motivational interviewing or behaviour change as core topics of study. The efficacy and importance of these concepts in making lifestyle changes is well documented [112, 113]. It is surprising, therefore, that a higher proportion of LM programmes do not explicitly mention these techniques. Research investigating the extent to which these techniques are employed across existing LM programmes would be useful.

### Barriers to LM Implementation

While some students are not receiving LM education in particular pillars, others are missing out entirely because it is not a mandatory topic in their university. Many of the programmes were offered as electives which offer a great opportunity for students to engage with the field of LM [36]. However, if the goal of LM is to drastically improve public health by shifting our society towards a more proactive, preventative form of medicine [114, 115], then this necessitates a collective effort from medical professionals [114, 116]. Including LM as a mandatory element of healthcare training might more effectively facilitate the engagement of all future healthcare practitioners in LM implementation.

Prior research has identified barriers that may be impeding such implementation. The primary barrier identified is the overloaded nature of medical curricula and related time constraints [48, 117, 118]. Mapping the existence of LM topics in current curricula has been suggested as a way of minimising the time burden of LM education implementation [48, 117, 118]. It is likely that LM topics are taught in many different disciplines and universities without explicitly being branded as LM, particularly as two pillars (nutrition and physical activity) are referenced in the USMLE content outline as part of ‘Lifestyle and routine preventative health care’ [119]. However, even so, it is unlikely that an adequate amount of educational time is spent on these topics. Prior research has shown that even nutrition, which is the most commonly taught pillar of LM, fails to reach the recommended amount of educational time of 25 h in most US universities [120]. The implementation of LM modules represents an opportunity for educational institutions to re-evaluate curricular content, checking if it aligns with the widely accepted public health goal of primary prevention, which also represents the basic principle of LM, while also comparing curricular content against educational recommendations. The Bar Ilan University [48] surveyed their entire pre-clinical curricula to identify lectures which already taught LM informally, to aid in formalisation of LM education in their university. This eliminated potential overlap and reduced the time constraints within an already packed curriculum. It also provides evidence that many aspects of LM are already taught in educational programmes and are simply not labelled as such. Formally recognising these interventions as a specific approach to healthcare may place an emphasis on these techniques which recommendations suggest, but which has not yet been achieved [2, 120–122]. This may encourage student engagement with LM topics. It also may encourage students to consider LM as a toolbox of possible interventions that should be considered for all patients, rather than only relating specific lifestyle interventions with specific interventions — for example, only considering sleep hygiene measures when dealing with a presentation of sleep apnoea. Varying student attitudes to the role of medical professionals in LM provision has been recognised as a barrier to LM implementation [48]. Essa-Hadad et al. surveyed 48 medical students on attitudes to LM and found broad disagreement on the statement ‘giving advice regarding healthy lifestyle is the role of allied healthcare professionals and not of the doctor’ [48]. Integrating LM across all aspects of healthcare education could help students recognise that LM should be practiced by all healthcare professionals.

Additional barriers to LM education implementation include, the requirement for external experts to teach LM [48, 117], the cost of hiring such experts [84], a lack of equal buy-in across faculties [84] and disagreement on the appropriateness of providing LM education during hospital-based placements [48]. Finally, many researchers have recognised assessment as a driver of learning, and suggest that LM is inadequately assessed by national medical boards such as the USMLEs [48, 117, 119, 123], which disincentivises its inclusion in curricula. These barriers may explain why teaching of LM in pre-registration degrees appears underdeveloped in many countries.

The variety of countries in the Lifestyle Medicine Global Alliance [124] suggests there is a widespread appetite for LM education, yet overcoming these barriers to implementation may take time. While research into the specific barriers to LM education outside the USA is lacking, it is probable that LM is currently a US-centric branch of medicine because the first LM institution (the American College of Lifestyle Medicine) was established in that country, with international bodies forming a decade later. The institutions which have successfully implemented LM, both in the USA and in other countries such as Israel, have made some useful suggestions as to how barriers to implementation might be overcome. This guidance may assist more countries in expanding LM education. In addition to mapping LM in existing curricula and including LM in medical assessments, research suggests that the development of student interest groups may be a useful way to foster advocacy for LM inclusion in curricula [117, 123]. Such advocacy may be necessary in the inception of LM as some may see it as a detraction from traditional clinical medicine [84].

### LM Education Across Healthcare Professions

While research articles contained within this review [56, 64, 73, 81, 84] suggest that there is a role for health professionals other than physicians in LM provision, there is a distinct lack of documented LM education for these professionals in training. Without widespread education of students in these disciplines, patients remain unlikely to receive LM advice outside of visits to their physician. The lack of multidisciplinary LM education (only one programme — Loma Linda University in 2024 — taught to more than one discipline) also contrasts with the wide variety of teaching staff providing LM education, which would suggest that each of these professions — such as psychologists, physiologists and social workers — has a role to play in the field of LM. Prior research has also emphasised the importance of multidisciplinary approaches to LM implementation [125–128]. Wetherill et al. have cited time constraints as a particularly pertinent barrier to LM education for physician associates, as an aim of this pre-registration degree is to help fill healthcare shortages by providing a degree that is shorter than the traditional medical degree. They suggest that adding additional content may hinder this aim [84]. However, other programmes that have introduced LM to physician associates have found it is a feasible change to make despite these restrictions [56, 73]. For other healthcare professions such as pharmacy and nursing, there is little research both about the barriers to LM education implementation and about possible solutions — further research in this area is required.

### Pedagogies Employed

The teaching methodologies used to provide LM education vary widely, and the data in this review serve as a reservoir of ideas which can be used for the construction of new LM education programmes. It also provides insight into the methodologies that existing programmes prioritise, such as lectures, cooking activities, group discussions, patient counselling, journalling and case studies.

The majority of programmes placed great emphasis on experiential learning and practical activities to engage learners and allow them to better contextualise the treatments that they will be prescribing [64, 129–131]. Reflection, personal goal setting, fitness tests, lifestyle assessments, lifestyle prescriptions, role-play and patient counselling were employed to give insight into what it is like to make and coach lifestyle changes — providing students with the experience of both patient and clinician [64, 132].

Bottcher et al. found that combining interactive lectures with cooking activities (used by 35% of programmes in the review) and discussion of recipes improved student LM counselling competencies, student knowledge of nutrition and student attitudes to LM counselling [39]. Malatskey et al. demonstrated that the LM counselling of patients by healthcare students can both increase students’ self-perceived LM competencies and improve patient lifestyle [62]. This provides evidence for the usefulness of patient counselling as a LM pedagogy. Polak et al. also showed that students had a positive view of such coaching activities, and that all faculty members surveyed (24) supported the initiative following its implementation. Brennan et al. found that goal setting (used by 21% of programmes in the review) was effective in changing student lifestyle behaviours in respect to stress management and nutrition but not physical activity — though the authors suggest a small sample size and large range of activity scores may have contributed to the lack of significant findings for physical activity [40]. Collectively, these studies suggest that incorporating practical elements to LM teaching is of value, though changes in student behaviour and attitudes may not translate into better patient outcomes, as is discussed further in the next section.

Most studies evaluated their programme’s ability to improve LM competencies but included many different pedagogies in these programmes and did not evaluate the efficacy of individual pedagogies. For example, Pasarica et al. show that a 90-min curriculum combining role-play, lifestyle histories and goal setting can improve student learning of LM interventions [71]. This supports the use of this combination of teaching methods in the delivery of LM content, but does not help to identify individual pedagogies that are effective. As LM education initiatives progress, it would be useful to consider and evaluate which pedagogies, or combination of teaching approaches, are most effective for teaching the different pillars of LM to ensure curricula are developed based on empirically supported approaches. Some previously validated approaches such as cooking activities for the nutrition pillar [133, 134] may also not be feasible in situations where factors such as time, facilities, student numbers and cost are prohibitive. It is therefore necessary to examine the effectiveness of various pedagogies to maximise the impact of LM in a variety of contexts.

### Course Evaluation

A wide variety of evaluation methods were also used for these programmes. Quantitative measurements ranged from measurements of anxiety to timing exercise to BMI and more. These quantitative measurements may however be more representative of a student’s own personal lifestyle changes, rather than their ability to counsel lifestyle changes for others. Some programmes used summative MCQs to assess student knowledge of lifestyle medicine, which may more objectively measure students’ knowledge of LM interventions. Many programmes used Likert-scales to allow students to rate their confidence in delivering, understanding of or knowledge of LM counselling before and after they receive the LM education. This aligns well with programme aims of LM intervention delivery, yet it does not offer an objective measurement of a students’ ability to provide LM counselling, nor the effectiveness of the programme in training them to do so. Interestingly, several programmes mention the use of OSCEs either as a teaching methodology or as a method of programme evaluation. OSCEs may offer a more objective measurement of a student’s ability to deliver LM counselling [135], which could then be used to evaluate the effectiveness of teaching practices. Research into the design and implementation of LM OSCEs would be useful. It is interesting that a slight majority of programmes studied (58%) did not report using summative assessments of knowledge/competency to evaluate their programme. Without such assessments it is difficult to objectively measure programme outcomes. If LM content is not included in summative assessments, it also may detract from the ‘seriousness’ of these programmes, as healthcare students are likely to focus their time and attention on modules which contain summative exams.

### Timing and Delivery of LM Education

The review also revealed the ways in which LM modules have been embedded thus far. Though certain universities preferred to place LM modules in earlier years, education was broadly split between all years when comparing between universities. This suggests that there is currently no consensus that LM education should be placed at a particular stage of pre-registration degrees.

Most universities have preferred a hybrid approach to programme synchronicity or run synchronous programmes, with fully asynchronous programmes being less common. The modality of delivery, in-person or online, was more variable and perhaps reflects new teaching practices that have emerged following the COVID-19 pandemic [136, 137]. Fully online teaching is now more common and acceptable, and the capabilities of institutions for providing content online has vastly improved [138, 139], allowing for teaching methodologies such as flipped classrooms, videos and online modules to become more prominent. Teaching frequency tended to vary with the length of the programme — with longer programmes more likely to be spread out across a semester or several years and shorter programmes more likely to be delivered in a block, though there were a few short programmes that were spread out across several very brief lessons. The three German universities, Gottingen, Brandenburg and Gieben [39] did offer both formats but did not analyse the difference between disseminated and block class frequencies. It would be useful to compare these contrasting styles of class provision in future research. While post-graduate programmes in LM provide qualifications in LM, it was surprising that only one programme in this review provided certification, particularly as most programmes were electives and not incorporated into degrees. Granting recognition for the completion of such educational activities might encourage more students to engage in LM. Research into the potential benefits of LM certification at a pre-registration level would provide useful insights. Alternatively, incorporating LM modules as mandatory aspects of healthcare curricula would also make scholarship of LM an inherent part of pre-registration healthcare degrees, making the distribution of separate LM certification unnecessary.

### Limitations

Limitations of this review include restriction of the search to articles using the term ‘Lifestyle Medicine’. It is recognised that many of the concepts of LM are being taught in educational institutions, either under alternate terms such as preventative medicine or behavioural medicine, or simply as conventional medical topics such as nutrition, within other modules. While the above terms encompass some concepts that differ from LM, there is potential overlap that would be worth exploring as the mapping of LM in medical curricula continues. Similarly, mapping of the presence of individual pillars of LM — present within medical curricula but not labelled as LM — is an important area for future research that was not explored in this review due to time constraints.

Additionally, translational resource constraints impose an English-language bias onto this review. There are a significant number of sister organisations of the Lifestyle Medicine Global Alliance which are based in countries where English is not the primary language (e.g. The Brazilian College of Lifestyle Medicine & The Korean College of Lifestyle Medicine) [124]. It is therefore likely that research on LM education has been published in these countries that was not included in this review and thus limits its scope. Future study into LM education research published in other languages, is warranted. The search was also restricted to the largest healthcare professions, and it is recognised that disciplines outside the scope of this review may have implemented LM education into their pre-registration degrees.

The heterogeneity of studies in this review presented a difficulty in succinctly summarising results. Some publications gave brief details about LM in several universities, others described specific programmes that occurred in certain years in individual universities and some described courses taught to pre-registration students directly by healthcare institutions rather than by universities. While presenting the findings by programme as in Table 1 (see online resource B) is the most accurate way of displaying results, it does not allow for easy comparison between locations, nor does it facilitate an easy comparison between universities. Table 3 (see online resource D) collates findings by university (and also details findings from programmes taught by healthcare institutions), yet many programmes were held in the same university in different years. Programmes may be direct updates of previous programmes also detailed in the review, or they might be similar but separate modules run by the same university. Such details were not described by the literature. This review therefore details the extent and nature of LM education that is reported to date but does not necessarily indicate the exact state of LM education currently — as the review does not indicate which programmes are still running in the format described within this review and which are obsolete. Additionally, there was significant variation in the entities described by the published literature. For example, 20 of the 52 programmes which did not specify an award were either mandatory or had a mandatory component. It is quite possible that these LM programmes were incorporated into the awarding of grades within the main degree, but the authors did not specify this. The review only reflects entities that were reported and many gaps in knowledge remain where these characteristics were not reported in published literature.

## Conclusions

While the research presented in this review contains valuable information for guiding the design of new LM curricula, it more pressingly reveals the imbalanced delivery of LM across the six pillars. Social connection, avoidance of risky substances and sleep are not commonly taught topics. Efforts should be made to improve their inclusion in LM programmes. Currently, some evaluations of LM programmes focus on changes in student lifestyle metrics, rather than student knowledge or skill acquisition. Such assessments should be supported by measurements that relate to student ability to deliver LM interventions to others, such as OSCEs. The review also shows that the extent of LM teaching outside the USA remains limited. Attempts should be made to map and identify the extent to which LM topics are taught in universities globally. The development of such programmes is urgently required to mitigate the rising economic and health burdens caused by chronic disease. The same is true for LM programmes catering to students of other health disciplines outside of medical schools. The evidence for the effectiveness of LM is plentiful, but to realise these benefits new healthcare professionals must be adequately trained to implement LM.

## Supplementary Information

Below is the link to the electronic supplementary material.

Supplementary file1 (DOCX 29 KB)

Supplementary file2 (DOCX 107 KB)

Supplementary file3 (DOCX 24 KB)

Supplementary file4 (DOCX 41 KB)

## Data Availability

All data from this review is provided in Online Resource B (Supplementary file 2).
