# Supplementary material for: Lifestyle Medicine Pillars and Pedagogies in Pre-registration Health Profession Degrees: A Scoping Review
Source: Med Sci Educ. 2025 Mar 17;35(3):1787–801. doi: 10.1007/s40670-025-02359-y (PMC12228630; doi:10.1007/s40670-025-02359-y)
Supplement: Supplementary file 2 — Supplementary file2 (DOCX 107 KB) [file 40670_2025_2359_MOESM2_ESM.docx]

Online resource B - Table 1. Data Extraction Results

Article: Lifestyle Medicine pillars and pedagogies in pre-registration health profession degrees: A scoping review.

Journal: Medical Science Educator

Authors: Jack Natin^1^ Muhammad Ahmad Ashfaque^1^ Anne Hickey^1^ Frank Doyle^1^ Maria Pertl^1^

Affiliation: *^1^ - RCSI University of Medicine and Health Sciences*

Corresponding author: Jack Natin – jacknatin23@rcsi.com

| **Author & Year** | **Country & University** | **Degree & Year of degree** | **Pillars of LM**  (Core topics named but outside conventional 8 pillars) | **Tutors profession** | **Nature of programme & Award** | **Teaching & assessment methodologies employed.** | **Synchronicity & Mode of content delivery** | **Frequency of classes & Hours of tutelage** | **Method of programme evaluation & aspect being assessed.** |
| --- | --- | --- | --- | --- | --- | --- | --- | --- | --- |
| Asano et al.  2021. [34] | USA.  West-Virginia School of Osteopathic Medicine. | Medicine. 3,4. | Nutrition, physical activity, stress management. | Clinical exercise physiologist, diabetes care specialist, chef. | Elective. Award not specified. | Video lectures, handouts, reading, quizzes, lectures, literature reviews, online modules, diabetes counselling, group discussions, exercise prescription, fitness tests (VO2 max, body composition, muscular strength/endurance, flexibility) , exercise demonstrations, field trip (shopping for groceries), yoga, mindfulness, cooking lab, clinical shadowing, group discussions. | Hybrid. Hybrid. | Classes all day, daily, for 2 weeks. 68 hours total. | Pre and post-programme Likert-scale survey of perceptions of topic and experience of programme, open-ended qualitative questions. |
| Balasubramaniam et al.  2021. [35] | Not specified. | Not specified. | Not specified. | Not specified. | Elective.  Award not specified. | Students coached LM to children. | Not specified. | Not specified. | Qualitative feedback & Likert scale surveys of confidence in knowledge, enjoyment of programme and relevance of content. |
| Bansal et al.  2023. [36] | USA.  Loma Linda University, School of Medicine. | Medicine. 1,2,3,4. | Nutrition, physical activity, stress management, sleep, social connection, avoidance of risky substances. | Not specified. | Elective or LM track.  Award not specified. | Journal Club, clinical shadowing, cooking activities, wellness tracking, plant-based lunches, presentation, reading, personal improvement project, group presentations, nutrition tracking and prescription. | Not specified. | Elective was daily for 2 weeks. 50 hours total. | Not specified. |
| Bansal et al.  2023. [36] | USA.  Warren Alpert Medical School, Brown University | Medicine. 3,4. | Nutrition, physical activity, stress management, sleep, social connection, avoidance of risky substances. | Not specified. | Elective.  Award not specified. | Clinical shadowing, movement therapy, yoga, mindfulness, cooking activities. | Not specified. | Daily for 2 weeks. Total hours not specified. | Not specified. |
| Bansal et al.  2023. [36] | USA.  University of Central Florida School of Medicine. | Medicine.  4. | Nutrition, physical activity, stress management, sleep, social connection, avoidance of risky substances. | LM boarded physician. | Elective.  Credits. | Online lectures, Clinical shadowing, lifestyle histories, lifestyle prescriptions, providing nutrition education to patients, breathing exercises with patients. | Synchronicity not specified.  Hybrid. | Daily for 2 or 4 weeks. Total hours not specified. | Not specified. |
| Bansal et al.  2023. [36] | USA.  University of California Irvine School of Medicine. | Medicine.  Pre-clinical years. | Nutrition | Chef, dietician, health coach. | Not specified. | Cooking activities, case studies, presentations. | Not specified. | Not specified. | Not specified. |
| Basha et al.  2021. [37] | Not specified. | Nursing.  Year not specified. | Nutrition, physical activity, sleep, stress management, avoidance of risky substances. | Not specified. | Not specified. | Student lifestyle modification goal, tracking & reflection. | Not specified. | 2 week-long assignment.  Total hours not specified. | Survey of student learning perceptions. |
| Bermejo & Stiegmann.  2020. [38] | USA.  Tulane University School of Medicine. | Medicine.  Year not specified. | Nutrition, Physical activity, sleep, (behaviour change). | Not specified. | Not specified. | Lectures, hands-on culinary medicine experiences. | Not specified. | Not specified. | Not specified. |
| Bermejo & Stiegmann.  2020. [38] | USA.  University of South Carolina | Medicine. 1,2,3,4. | Nutrition, Physical activity, sleep, (behaviour change). | Not specified. | Not specified. | Lectures. | Not specified. | Frequency not specified. 86 hours total. | Not specified. |
| Bottcher et al.  2023. [39] | Germany.  University of Gottingen, Brandenburg Medical School, University of Gieben. | Medicine.  All years. | Nutrition. | Dietician, chefs, previous students of programme, physician. | Elective.  Award not specified. | Interactive lecture, recipe discussion, cooking activities, tasting sessions. | Synchronous.  Online in 2021, in-person in 2022. | Once a week for 7 weeks or as block of 4-5 days.  28 hours total. | Self-assessment questionnaires on counselling knowledge, attitudes and competencies, Likert-scale habit assessment, WHO-5 wellbeing index MCQs, all pre and post-programme. |
| Brennan et al.  2024. [40] | USA.  University of Toledo. | Medicine.  1,2. | Nutrition, physical activity, stress management. | Dietician & others. | Elective.  Course credit. | Lecture, student SMART goal-setting task, mindfulness, cognitive restructuring, breathing exercises, mindful eating, case studies, treatment plans. | Synchronicity not specified.  In-person, though online in 2020. | At least 5 group meetings.  Total hours not specified. | Pre and post screening tests: GAD-7, fruit and vegetable screening assessment, perceived stress scale, PHQ9, minutes of physical activity, steps, dietary fat. Likert rating of satisfaction with programme. |
| Curry.  2017. [42] | Israel.  Hadassah-Hebrew University School of Medicine | Medicine.  Year not specified. | Not specified. | Not specified. | Not specified. | Lectures, case studies, group discussions, experiential work, health coaching (friends in pre-clinical years, patients in clinical years), clinical shadowing. | Not specified. | Not specified. | Student and faculty satisfaction with curriculum. Frequency and content of student-student health-coaching interactions. |
| Drost et al.  2021. [43] | USA.  Mayo Clinic Alix School of Medicine in Arizona. | Medicine.  1. | Nutrition, Physical activity, stress management, sleep. | Multidisciplinary clinicians. | Mandatory. Award not specified. | Flipped classroom (online module + discussions), videos, lifestyle self-assessment, client coaching, didactics, small group discussions, label reading, virtual grocery store tour, diet prescription, meal plan with budget, documentaries with accompanying discussions, food tasting, fitness tests, exercise prescriptions, stress self-assessment, mindfulness practice. | Hybrid.  Hybrid. | Programme spread over 3 days.  11 hours total. | Pre and post-programme Likert-scale survey assessing knowledge of and attitude towards topics. Open-ended qualitative questions. |
| Dupuis et al. 2021. [44] | USA.  University of Central Florida College of Medicine. | Medicine.  3, 4. | Nutrition, physical activity, sleep, stress management, avoidance of risky substances, social connection. | LM certified practitioner. | Elective & Mandatory elements. | Didactics, reading, case-based learning, reflections, student goal-setting, clinical shadowing, group discussions, audience response activities, teaching patients, lifestyle assessments, lifestyle prescriptions, MCQs, OSCE. | Not specified. | Mandatory hours not specified. 4 week elective with unspecified hours. | Not specified. |
| Edens et al.  2018. [45] | USA.  Feinberg School of Medicine, Northwestern University. | Medicine.  1. | Nutrition (health coaching, behaviour change) | Not specified. | Not specified. | Coaching elementary students in nutrition. An external culinary medicine program. | Not specified. | Not specified. | Not specified. |
| Edge et al.  2024. [46] | USA.  Keck School of Medicine (KSOM) of University of Southern California (USC) . | Medicine.  1. | Nutrition, physical activity. | Not specified. | Mandatory within primary care track.  Award not specified. | Lecture, videos, reading, teaching exercise classes to local communities. | Synchronicity not specified. Hybrid. | 1 hour lecture & 35 minutes teaching exercise classes to patients. | Post-survey with closed and open-ended questions relating to perceived knowledge, confidence and satisfaction. |
| Essa-Hadad et al.  2015. [47] | Israel.  Bar Ilan University. | Medicine.  Year not specified. | Not specified. | Public Health tutors. | Mandatory.  Award not specified. | Lectures, cooking demonstration, exercise activity, yoga class, application of project in local community, small group discussions, MCQs. | Not specified. | All hours in 1 day.  6 hours total. | Likert scale survey of perceptions of programme, oral feedback on programme. |
| Essa-Hadad et al.  2022. [48] | Israel.  Bar Ilan University. | Medicine.  1,2,3,4. | Nutrition, physical activity, stress reduction, reduction of avoidance of risky substances, sleep, sexual health, (behaviour change). | Not specified. | Mandatory. Incorporated into main degree. | Lectures, counselling, patient coaching, yoga, exercise practical, cooking activities, group discussions, mindfulness sessions, case-based learning, role play with actor. | Not specified. | Scattered throughout the 4 year medical degree. 58 hours total. | Online questionnaire relating to self-perceived competence, attitudes and confidence. |
| Frame.  2021. [49] | USA.  George Washington School of Medicine and Health Sciences. | Medicine.  Year not specified. | Nutrition, physical activity. | Credits. | Not specified. | Patient history taking and lifestyle prescriptions. | Not specified. | Two 3 credit-hour programmes.  6 hours total. | Not specified. |
| Frates et al.  2016. [50] | USA.  Harvard Medical School. | Medicine.  1,2. | Nutrition, physical activity, (motivational interviewing). | Physician. | Elective.  No award. | Lecture. | Synchronous.  In-person. | One hour long lecture. | Pre and post-programme survey of knowledge, attitudes and confidence regarding lifestyle medicine, including multiple choice questions. |
| Harvey et al.  2022. [51] | UK.  Imperial College School of Medicine in London. | Medicine.  1,2. | Nutrition, physical activity, sleep, (mental health). | Clinicians. | Mandatory (incorporated into main degree).  15% of learning and assessment. | Flipped classroom, small group tutorials, reflections. | Not specified. | 15% of learning and assessment. | Questionnaire – Likert-scale and open-ended questions relating to importance of lifestyle medicine. |
| Heiman et al.  2018. [52] | USA.  Northwestern University Feinberg School of Medicine. | Medicine.  1,2,3,4. | Nutrition, physical activity (behaviour change) | Not specified. | Mandatory.  Incorporated into main degree as one of 5 curricular threads. | LM thread: personal behaviour change plan, self-reflection, written examination, scholarly project, history taking, lectures, prescribing, OSCEs, small group assessments. | Not specified. | Not specified. | Traditional measures of knowledge and clinical performance. Longitudinal portfolio. |
| Humerick et al.  2024. [53] | USA.  West Virginia University School of Medicine | Medicine.  1,2,3,4. | Nutrition, physical activity, stress management, sleep | Psychiatrist, paediatrician, cardiologist, internal medicine physician, endocrinologist, exercise physiologist, behavioural medicine psychologist, chef, culinary medicine certified family medicine physician. | Optional degree track.  Track qualification and optional culinary medicine certificate. | Lectures, readings, quizzes, cooking activities, journal clubs, clinical learning groups, summer externships, clinical electives, capstone project, elective culinary medicine certification. | Synchronicity not specified.  Hybrid. | Frequency not specified. 300 hours total over 4 years. | Qualitative and quantitative pre and post-programme surveys relating to perceptions of attendance, projects and the Certified Culinary Medicine Specialists exam. Number of students completing the Certified Culinary Medicine Specialists exam, number of students applying to track, number of students completing the final project. |
| Kay & Pasarica.  2019. [54] | USA.  University of Central Florida. | Medicine.  2. | Not specified. | Not specified. | Either elective or mandatory. Awards not specified. | lectures, small group sessions, problem solving clinical scenarios, peer to peer feedback. | Either synchronous or asynchronous (not hybrid).  Either online or in person (not hybrid) | Not specified. | Attendance, assignment completion, oral feedback, faculty subjective satisfaction, grading of quality of student engagement. |
| Kaye et al.  2019. [55] | USA.  Wake Forest School of Medicine. | Medicine.  1. | Nutrition, physical activity, stress management, sleep. | Student-led. | Elective.  Award not specified. | Building a balanced plate exercise, muscle and bone building exercises, grocery shopping challenges, budget kitchen building exercise, food diaries, sleep diaries. | Not specified. | Contained within programme.  Total hours not specified. | Not formally evaluated. |
| Keyes & Gardner.  2020. [56] | USA.  Not specified. | Physician associates.  1. | Nutrition, physical activity, stress management, avoidance of risky substances (as smoking cessation). | LM certified faculty member. | Elective.  Award not specified. | Lifestyle history and lifestyle prescription, guideline learning. | Not specified. | 4 classes of 50 minutes.  3 hours and 20 minutes total. | Survey of self-perceived competency pre and post programme. Prevention and lifestyle assessment write-up. Formally assessed critical thinking session. |
| Krpalek.  2024. [57] | USA.  Loma Linda university. | Students in school of allied health professions.  All years. | Nutrition, physical activity, stress, social connection. | Occupational therapist, doctor. | Elective.  Entered into raffle for 50 dollar gift card. | 5 min video, list of resources, interactive activities, student goal-setting, diet reflection, motivation and reward identification, exercise prescription, mindfulness practice, journalling. | Asynchronous.  Online. | 5 minutes per week for 4 weeks.  20 minutes total. | Participants completed the Depression, Anxiety, and Stress Scale (DASS-21) and the Health-Promoting Lifestyle Profile II (HPLP-II) before and after the program. Additionally, they completed a 7-item post-program survey and engaged in semi-structured interviews approximately one month post participation. |
| Kushner & Van Horn.  2018. [58] | USA.  Northwestern University Feinberg School of Medicine. | Medicine.  1,2. | Nutrition, physical activity, avoidance of risky substances sleep, stress (behaviour change, body weight). | Not specified. | Mandatory. Incorporated into other modules in main degree. | Taught throughout other modules. Behaviour change plan 6 week activity. MCQs, group discussions, OSCEs. | Not specified. | 81 sessions spread over 2 years. 81 hours total. | Not specified. |
| Kushner et al.  2014. [59] | USA.  Northwestern University Feinberg School of Medicine. | Medicine.  1,2. | Nutrition, physical activity, smoking, (body weight). | Not specified. | Mandatory. Incorporated into other modules in main degree. | Nutrition focused history and exam, cardiopulmonary fitness measurements, 10 week behaviour change plan | Not specified. | Not specified. | Not specified. |
| Lee et al.  2023. [60] | USA.  University of Texas Southwestern Medical School. | Medicine.  1,2,3,4. | Nutrition (others informally). | Not specified. | Nutrition elective, LM informally integrated into main degree.  Award not specified. | Lectures. | Taught within other modules. | Not specified. | Survey relating to student perceptions of LM and health behaviours. |
| Lee et al.  2023. [60] | USA.  Texas College of Osteopathic Medicine. | Medicine.  Year not specified. | Nutrition. | Not specified. | Nutrition elective.  Award not specified. | Not specified. | Not specified. | Not specified. | Survey relating to student perceptions of LM and health behaviours. |
| Lisevick.  2023. [61] | USA.  Quinnipiac university. | Medicine.  Pre-clinical years. | Nutrition, physical activity, sleep, stress management, avoidance of risky substances, social connection. | Faculty & alumni. | Elective.  Award not specified. | Case studies, workshops, guest speakers, book clubs, reflections, receiving coaching from programme alumni. | Not specified. | 2/3 hour-long sessions over 16 weeks.  2-3 hours. | Pre and post-programme self-assessment questionnaire with Likert-scale questions. |
| Malatskey et al.  2022. [62] | Israel.  Bar Ilan University. | Medicine.  1,2. | Nutrition, (behaviour change, motivational interviewing). | Physicians | Elective.  Award not specified. | Case presentations, patient coaching (over 18 months), didactics, simulating clinic with actors, reading food labels, food diary. | Not specified. | 6 sessions. 4 hours and 30 minutes total. | Likert questionnaire and qualitative feedback (from a focus group) related to student perceived self-efficacy and health behaviours. |
| Malatskey et al.  2019. [63] | Israel.  Bar Ilan University. | Medicine.  1. | Nutrition, physical activity, stress, sleep, avoidance of risky substances [as smoking cessation], (motivation). | Not specified. | Mandatory. Incorporated into other modules in main degree. | Lectures, cooking activities, exercise, yoga. | Not specified. | 1 day at the beginning of 1st year and 2 days at the end of 1st year. 24 hours total. | Pre and post-programme Likert questionnaire and qualitative feedback relating to student attitudes towards LM. |
| Mattison & Nemec.  2014. [64] | USA.  Western New England University. | Pharmacy.  3. | Nutrition, physical activity, sleep, stress management, avoidance of risky substances, (motivational interviewing). | Not specified. | Elective.  3 credits. | Student goal-setting and completing, reflections, relaxation videos, muscle relaxation exercises, yoga, taste-testing organic vs non-organic foods, colour therapy, meditation, financial wellness planning, stretching, pet therapy, mindfulness, lectures, quizzes, food diaries, patient cases, role-playing, designing patient care plans, composite lifestyle index tool, fitness testing, group presentations. | Not specified. | Twice a week for 15 weeks.  60 hours total. | Pre and post-programme survey on perceptions of LM and confidence in implementing LM interventions, pre and post-programme fitness test, examinations assessing concept comprehension. |
| McGrady et al.  2021. [66] | USA.  University of Toledo. | Medicine.  1,2. | Nutrition, physical activity, stress management. | Dietician, others. | Elective.  Course credit. | Lectures, case studies, personal lifestyle assessment, mindfulness, cognitive restructuring, breathing exercises, mindful eating,  treatment plans, student SMART goal-setting task. | Synchronicity not specified.  Online or in-person. | 5 minutes per week for 4 weeks.  20 minutes total. | Pre and post-programme screening tests: GAD-7, fruit and vegetable intake questionnaire, perceived stress scale, PHQ9, minutes of physical activity, steps, dietary fat. Likert scale rating usefulness of course and progress towards lifestyle goal achievement. |
| McGrady et al.  2019. [65] | USA.  University of Toledo. | Medicine.  1,2. | Nutrition, physical activity, stress management, avoidance of risky substances (as smoking cessation). | Dietician, faculty physicians. | Elective.  Course credit. | Lectures, student SMART goal setting task, mindfulness, cognitive restructuring, breathing exercises, focus groups, case-based discussions. | Synchronous.  In-person. | 7 sessions over one semester.  5 hrs 35 minutes total. | Pre and post-programme screening tests: GAD-7, fruit and vegetable intake questionnaire, PHQ9, minutes of physical activity. Likert scale rating usefulness of programme. |
| Muscato et al.  2018. [67] | USA.  Ohio University. | Medicine.  Year not specified. | Nutrition. | Not specified. | Not specified. | Video, articles, lectures, clinical shadowing. | Not specified. | Frequency not specified.  2 hours total. | Not specified. |
| Muscato et al.  2018 [67] | USA.  University of Toledo. | Medicine.  1,2. | Nutrition, physical activity, stress management. | Not specified. | Elective.  Award not specified. | Group discussions, case studies, lectures. | Not specified. | Not specified. | Not specified. |
| Muscato et al.  2018 [67] | USA.  Virginia tech Carilion school of medicine. | Medicine.  3. | Nutrition, physical activity, stress management, avoidance of risky substances, sleep, social connection. | Physician, public health educator, exercise physiologist. | Not specified. | Lectures, experiential learning, behaviour diary, case studies. | Not specified. | Frequency not specified.  3 hours total. | Measurements of student confidence in LM prescriptions. |
| Muscato et al.  2018 [67] | USA.  Western University of Health Sciences College of Osteopathic Medicine of the Pacific. | Medicine.  All years. | Nutrition, physical activity, stress management, avoidance of risky substances [as smoking cessation], (weight loss, self-care, mindfulness). | Not specified. | Mandatory & elective components.  Award not specified. | Community service. | Not specified. | Frequency not specified.  8 hours total. | Not specified. |
| Muscato et al.  2018 [67] | USA.  University of South Carolina. | Medicine.  1,2,3,4. | Not specified. | Dieticians, exercise physiologists, wellness coaches. | Mandatory.  Award not specified. | Case studies, OSCEs, reflections. | Not specified. | Not specified. | Not specified. |
| Nguyen et al. 2023. [68] | USA.  Edward Via College of Osteopathic Medicine Carolinas. | Medicine.  2. | Nutrition, physical activity. | Physicians, chefs, dieticians. | Elective.  Award not specified. | Lectures, guest speakers, recipe creation, food tasting, cooking activities, volunteering at food initiatives. | Synchronicity not specified.  Hybrid. | 2 hours a month for 8 months.  16 hours total. | Pre and post-programme Likert questionnaire on confidence, competency and satisfaction. Monthly qualitative surveys of knowledge and confidence. |
| Ogawa et al.  2023. [69] | USA.  University of Rochester, School of Medicine and Dentistry. | Medicine.  3,4. | Nutrition, stress management, physical activity, (Informally: sleep, social connection and substance use) | LM practitioners. | Elective.  Award not specified. | Clinical observation, reading, patient coaching, plant diet challenge, student SMART goal setting task, meditation, walking meetings. | Synchronicity not specified.  Hybrid. | 2 week block of classes.  Total hours not specified. | Post-programme questionnaire on understanding of LM and confidence implementing LM in practice. Evaluation of knowledge and skills by preceptors. |
| Pasarica & Kay.  2020. [70] | USA.  University of Central Florida. | Medicine.  3. | Nutrition, physical activity, avoidance of risky substances [as smoking]. | Not specified. | Not specified. | Online learning module, role-play, case studies. | Hybrid.  Hybrid. | Once a week for 12 weeks. 3 hours total. | Kirkpatrick pyramid, Likert-scale questionnaire of student satisfaction, summative OSCE. |
| Pasarica et al.  2019 [71] | USA.  University of Central Florida. | Medicine.  3. | Individual pillars not specified, (Motivational interviewing) | Physician & psychologist | Mandatory.  Award not specified. | Role-playing, SMART goals, online learning module, small group discussions. | Hybrid.  Hybrid. | Classes spread throughout 12 week module. 1.5 hours total. | Likert questionnaire of student perceptions of learning, Kirkpatrick pyramid, scoring of SMART goals, engagement measuring. |
| Pasarica & Topping.  2017. [72] | USA.  University of Central Florida. | Medicine.  2. | Nutrition, physical activity | “Lifestyle Medicine expert” | Award not specified. | Reading, clinical scenarios, small group discussions, presentation. | Not specified. | 1 session.  2 hours total. | Pre and post-programme Likert-scale questionnaire regarding confidence implementing LM in practice. |
| Phillips et al.  2021. [73] | USA.  VA Boston healthcare system (not a university). | Physician associates.  1,2,3. | Nutrition, physical activity, stress reduction, (motivational interviewing). | Clinicians, physiotherapists, certified health coach. | Mandatory.  None. | Didactics, clinical observation, personal self-care activities, exercise prescriptions, cooking activities, presentation, reflection. | Hybrid.  Hybrid. | Classes distributed across 5 weeks. 12 hours. | Multiple choice quiz, self-efficacy questionnaire. |
| Polak et al.  2017. [74] | Israel.  Hadassah Hebrew University. | Medicine  1,2,3,4,5,6. | Nutrition, physical activity, avoidance of risky substances [as smoking cessation] (behavioural change). | GPs, sports physicians, exercise physiologists, health psychologists, public health physicians, social workers. | 3 mandatory modules, one elective module.  Award not specified. | Case presentations, patient coaching, lectures, history taking, discussions, webinars, bedside teaching, online module, coaching friends/relatives. | Hybrid.  Hybrid. | Classes distributed across 6 years.  58 hours total. | Pre and post-programme questionnaires with Likert-scale questions assessing students self-perceived ability to deliver LM interventions, open-ended questions. |
| Poulton et al.  2022. [75] | USA.  University of North Carolina. | Medicine.  3,4. | Nutrition, sexual health. | Not specified. | Elective.  Award not specified. | Lectures, small group discussions, cooking activities, online modules. | Hybrid.  Online. | Not specified. | Pre and post-programme surveys regarding confidence implementing LM in practice. |
| Razavi et al.  2023. [76] | USA.  ‘Health meets food curriculum’ – many universities. | Medicine.  Year not specified. | Nutrition. | Physicians, dieticians, chefs. | Mandatory in some universities, elective in others.  Award not specified. | Nutrition counselling, cased based learning, cooking activities, didactics, discussions | Synchronous.  Either online or in-person (not hybrid). | One 4 hour session per week for 8 weeks. 32 hours total. | Pre and post-programme survey with Likert-scale questions relating to student attitudes, habits and self-perceived counselling competencies. MedDiet score. |
| Rea et al.  2021. [2] | USA.  Harvard Medical school. | Medicine.  Year not specified. | Not specified. | Not specified. | Elective.  Award not specified. | Not specified. | Not specified. | Not specified. | Not specified. |
| Rea et al.  2021. [2] | USA.  University of Oklahoma. | Medicine.  1,2. | Not specified. | Not specified. | Mandatory.  Award not specified. | Didactic, experiential, meditation, mindfulness, cooking activities, community outreach, simulated patients, research, community class facilitation. | Not specified. | Frequency not specified.  69 hours total. | Not specified. |
| Rea et al.  2021. [2] | USA.  A.T. Still University. | Medicine.  1,2,3,4. | Not specified. | Not specified. | Mandatory.  Award not specified. | Lifestyle coaching, cooking activities, mindfulness, student goal-setting task. | Not specified. | Frequency not specified.  More than 80 hours total. | Not specified. |
| Rea et al.  2021. [2] | USA.  University of South Carolina. | Medicine.  Year not specified. | Not specified. | Chef, “Lifestyle Medicine expert”. | Both.  Award not specified. | Case studies, cooking activities, nutrition reports, plate presentations, patient interactions | Not specified. | Frequency not specified.  More than 80 hours total. | Not specified. |
| Rea et al.  2021. [2] | USA.  Loma Linda University. | Medicine.  1,2,3,4. | Not specified. | Not specified. | Both.  Award not specified. | Not specified. | Not specified. | Frequency not specified.  More than 80 hours total. | Not specified. |
| Rockfeld et al.  2020. [77] | USA.  Quinnipac university. | Medicine.  3. | Nutrition, physical activity, stress management. | Physicians. | Mandatory.  Award not specified. | Didactics, reflection, draw dinner plate, group discussions, deep breathing activity, walk, presentation. | Synchronous.  In-person. | 3 workshops of 1 hour each.  3 hours total. | Pre and post-programme Likert survey regarding self-perceived knowledge, skills and attitudes towards LM. |
| Sadiq et al.  2024. [78] | Pakistan.  Riphah international university. | Medicine.  1,2,3. | Nutrition, physical activity, sleep, stress management, avoidance of risky substances, social connections (behaviour change, positive psychology, empowering self and others). | Not specified. | Mandatory.  Award not specified. | Lectures, problem based learning, skill labs, history taking, clinical rotations, case studies, MCQs, OSCEs, VIVA, research projects. | Not specified. | Not specified. | Not specified. |
| Shehade & Broughton.  2024. [79] | USA.  Northeastern University Bouvé College of Health Sciences. | Physician associates.  Year not specified. | Nutrition, physical activity, sleep, stress management, avoidance of risky substances, social connection. | Not specified. | Mandatory.  Award not specified. | Lectures, videos, meditation, breathing exercises, lifestyle prescriptions. | Not specified. | 8 lectures.  Hours not specified. | Not specified. |
| Sinha et al.  2023. [80] | USA.  University of South Carolina. | Medicine.  1,2,3,4. | Not specified. | Not specified. | Not specified. | Not specified. | Not specified. | Frequency not specified.  More than 80 hours total. | Not specified. |
| Sinha et al.  2023. [80] | USA.  University of Oklahoma. | Medicine.  1,2. | Nutrition, sleep, physical activity, stress management. | Not specified. | Both.  Award not specified. | Meditation, mindfulness, culinary medicine, community outreach, simulated patients. | Not specified. | Not specified. | Not specified. |
| Sinha et al.  2023. [80] | USA.  Harvard Medical School. | Medicine.  3,4. | Not specified. | Not specified. | Elective  Award not specified. | Lectures, workshops. | Not specified. | Not specified. | Not specified. |
| Sinha et al.  2023. [80] | USA.  Stony brook university. | Medicine.  Year not specified. | Nutrition, physical activity, sleep, stress management. | Family physician, dietician, social worker, psychiatrist. | Both.  Award not specified. | Lectures, literature reviews, discussion groups, cooking activities, presentations, guideline reviews, clinical observations, role-playing, field trips. | Not specified. | Not specified. | Not specified. |
| Stauffer et al.  2022. [81] | USA.  VA Boston healthcare system (not a university). | Physician associate.  1,2,3. | Not specified. | Not specified. | Not specified. | Not specified. | Not specified. | Distributed across 5 weeks. Total hours not specified. | Clinical vignettes pre and post programme. |
| Trilk et al.  2019. [82] | USA.  University of South Carolina. | Medicine.  1,2,3,4. | Nutrition,, physical activity, stress management, sleep. | Not specified. | Mandatory.  Award not specified. | Lectures, problem based learning, case based learning, simulated patients, role play, clinical experiences, exercise prescriptions, dietary counselling, behavioural change counselling (motivational interviewing), cooking activities. | Not specified. | Classes distributed throughout 4 years.  86.5 hours total. | Summative exam performance statistics. Student evaluations of faculty. |
| University of South Carolina, Greenville School of Medicine website.  2024 [41] | USA.  University of South Carolina. | Medicine.  1,2,3,4. | Nutrition, physical activity (behaviour change, self-care). | Chefs and teaching faculty. | Mandatory. Incorporated into other modules in main degree. | Planting crops, cooking activities, bike rides. | Not specified. | Not specified. | Not specified. |
| Visaria et al.  2022. [83] | Not specified. | Not specified. | Not specified. | Not specified. | Not specified. | Online module, simulated patient assessment, supervised community screening, blood pressure measurements, lifestyle history, lifestyle counselling. | Synchronicity not specified.  Hybrid. | Not specified. | Content-based MCQ pre and post-training. |
| Wetherill et al.  2019. [84] | USA.  University of Oklahoma & University of Tulsa. | Physician associate.  2. | Nutrition, physical activity, avoidance of risky substances (body weight reduction). | Physician, dietician, chef. | Elective/mandatory nature not specified.  2 credits. | Didactics, cooking activities, case studies, quizzes, reflections, online modules, group project. | Hybrid.  Hybrid. | 4 weeks.  2 credit hours. | Pre and post-programme student perceptions of knowledge and confidence, qualitative open-ended questions relating to experience of programme, MedDiet score, BMI. |
