# Supplementary material for: Lifestyle Medicine Pillars and Pedagogies in Pre-registration Health Profession Degrees: A Scoping Review
Source: Med Sci Educ. 2025 Mar 17;35(3):1787–801. doi: 10.1007/s40670-025-02359-y (PMC12228630; doi:10.1007/s40670-025-02359-y)
Supplement: Supplementary file 3 — Supplementary file3 (DOCX 24 KB) [file 40670_2025_2359_MOESM3_ESM.docx]

Online Resource C – Table 2. The frequency of LM education characteristics in universities and in educational programmes

Article: Lifestyle Medicine pillars and pedagogies in pre-registration health profession degrees: A scoping review.

Journal: Medical Science Educator

Authors: Jack Natin^1^ Muhammad Ahmad Ashfaque^1^ Anne Hickey^1^ Frank Doyle^1^ Maria Pertl^1^

Affiliation: *^1^ - RCSI University of Medicine and Health Sciences*

Corresponding author: Jack Natin – jacknatin23@rcsi.com

|  | **Number of universities.** | **Percentage of universities.** *(rounded to the nearest whole number) (calculated from total universities/programmes that specified data)* | **Number of programmes.** | **Percentage of programmes.**  *(rounded to the nearest whole number) (calculated from total universities/programmes that specified data)* |
| --- | --- | --- | --- | --- |
| **Health profession degree being taught:** |  |  |  |  |
| Medicine | 36 | 97 % | 59 | 87 % |
| Physician associates | 3 | 8 % | 6 | 9 % |
| All allied health professions | 1 | 3 % | 1 | 1 % |
| Pharmacy | 1 | 3 % | 1 | 1 % |
| Nursing | 0 | 0 % | 1 | 1 % |
| *Not specified* | *0* |  | *3* |  |
| **Degree year being taught:** |  | | | |
| 1st | 20 | 69 % | 38 | 69 % |
| 2nd | 20 | 69 % | 37 | 67 % |
| 3rd | 22 | 76 % | 31 | 56 % |
| 4th | 18 | 62 % | 24 | 44 % |
| 5th | 1 | 3 | 1 | 2 |
| 6th | 1 | 3 | 1 | 2 |
| ‘Pre-clinical’ | 2 | 7 | 2 | 4 |
| *Not specified* | *8* |  | *16* |  |
| **Faculty profession.** |  | | | |
| Physician/Doctor | 15 | 65 % | 15 | 44 % |
| Dietician | 10 | 43 % | 13 | 38 % |
| Chef | 10 | 43 % | 12 | 35 % |
| Other | 6 | 26 % | 9 | 26 % |
| LM Boarded physician / LM expert | 4 | 17 % | 7 | 21 % |
| Exercise physiologist | 5 | 22 % | 5 | 15 % |
| Alumni | 5 | 22 % | 5 | 15 % |
| Clinician (practice unspecified) | 2 | 9 % | 3 | 9 % |
| Public health educator | 3 | 13 % | 3 | 9 % |
| Psychologist | 4 | 17 % | 3 | 9 % |
| Social worker | 2 | 9 % | 2 | 6 % |
| Diabetes specialist | 1 | 4 % | 1 | 3 % |
| *Not specified* | *14* |  | *37* |  |
| **Nature of LM instruction** |  | | | |
| Elective | 14 | 44 % | 26 | 46 % |
| Mandatory | 7 | 22 % | 19 | 34 % |
| Both elective & Mandatory | 11 | 34 % | 11 | 20 % |
| *Not specified* | *5* |  | *15* |  |
| **Programme Award** |  | | | |
| Credit | 6 | 46 % | 8 | 42 % |
| Part of main degree | 5 | 38 % | 8 | 42 % |
| *Not specified* | *24* |  | *52* |  |
| **Teaching Methodology** |  | | | |
| Other | 27 | 75 % | 41 | 62 % |
| Lectures / Didactics | 27 | 75 % | 41 | 62 % |
| Cooking activities | 16 | 44 % | 23 | 35 % |
| Group discussions | 15 | 42 % | 21 | 33 % |
| Case studies | 12 | 34 % | 18 | 27 % |
| Patient counselling/coaching | 11 | 31 % | 17 | 26 % |
| Journaling / reflection | 12 | 34 % | 16 | 24 % |
| Clinical shadowing | 11 | 31 % | 14 | 21 % |
| Lifestyle prescription | 10 | 28 % | 14 | 21 % |
| Mindfulness/meditation | 11 | 31 % | 14 | 21 % |
| Student goal-setting/Behaviour change plan | 7 | 19 % | 13 | 21 % |
| Fitness tests/exercise practicals | 9 | 25 % | 11 | 17 % |
| Role-play | 6 | 17 % | 11 | 17 % |
| Presentations | 9 | 25 % | 10 | 15 % |
| Online module | 6 | 17 % | 9 | 14 % |
| Lifestyle history | 5 | 14 % | 8 | 12 % |
| Food tasting / Mindful eating | 7 | 19 % | 8 | 12 % |
| Quizzes/MCQs | 6 | 17 % | 7 | 11 % |
| Reading | 7 | 19 % | 7 | 11 % |
| Videos | 7 | 19 % | 7 | 11 % |
| Yoga | 4 | 11 % | 6 | 9 % |
| Breathing exercises | 4 | 11 % | 5 | 8 % |
| OSCE (Objective Structured Clinical Examination) | 4 | 11 % | 4 | 6 % |
| Unspecified experiential learning | 3 | 8 % | 3 | 5 % |
| Lifestyle self-assessment | 3 | 8 % | 3 | 5 % |
| *Not Specified* | *1* |  | *5* |  |
| **Synchronicity of lesson** |  | | | |
| Synchronous | 7 | 50 % | 8 | 42 % |
| Asynchronous | 2 | 14 % | 2 | 11 % |
| Hybrid | 7 | 50 % | 9 | 50 % |
| *Not specified* | *23* |  | *53* |  |
| **Modality of lesson** |  | | | |
| Online | 7 | 39 % | 8 | 31 % |
| In-person | 7 | 39 % | 10 | 39 % |
| Hybrid | 10 | 55 % | 14 | 55 % |
| *Not specified* | *19* |  | *45* |  |
| **Frequency of classes** |  | | | |
| Disseminated | 20 | 77 % | 27 | 66 % |
| Block delivery | 14 | 54 % | 17 | 42 % |
| *Not specified* | *11* |  | *30* |  |
| **Programme assessment methodology** |  | | | |
| Likert scale / ratings | 17 | 63 % | 30 | 75 % |
| Qualitative questions | 17 | 63 % | 23 | 58 % |
| Quantitative assessments | 17 | 63 % | 21 | 53 % |
| *Not specified* | *10* |  | *31* |  |
| **Summative assessment of knowledge/competencies** |  |  |  |  |
| Yes – there is a summative assessment | 11 | 41 % | 20 | 42 % |
| No – there is no summative assessment | 16 | 59 % | 28 | 58 % |
| *Not specified* | *10* |  | *23* |  |
