# Supplementary material for: Lifestyle Medicine Pillars and Pedagogies in Pre-registration Health Profession Degrees: A Scoping Review
Source: Med Sci Educ. 2025 Mar 17;35(3):1787–801. doi: 10.1007/s40670-025-02359-y (PMC12228630; doi:10.1007/s40670-025-02359-y)
Supplement: Supplementary file 4 — Supplementary file4 (DOCX 41 KB) [file 40670_2025_2359_MOESM4_ESM.docx]

Online resource D - Table 3. The attributes of LM education that have been offered in each university.

Article: Lifestyle Medicine pillars and pedagogies in pre-registration health profession degrees: A scoping review.

Journal: Medical Science Educator

Authors: Jack Natin^1^ Muhammad Ahmad Ashfaque^1^ Anne Hickey^1^ Frank Doyle^1^ Maria Pertl^1^

Affiliation: *^1^ - RCSI University of Medicine and Health Sciences*

Corresponding author: Jack Natin – jacknatin23@rcsi.com

| **University & Country** | **Degree being taught & Year of degree** | **Pillars of LM**  (Core topics named but outside conventional 8 pillars) | **Tutors profession** | **Nature of programme & Award** | **Teaching & assessment methodologies employed.** | **Synchronicity & Mode of content delivery** | **Frequency of classes & Hours of tutelage** | **Method of programme evaluation & aspect being assessed.** |
| --- | --- | --- | --- | --- | --- | --- | --- | --- |
| A.T. Still University.  USA. [2] | Medicine.  1,2,3,4. | Not specified. | Not specified. | Mandatory.  Award not specified. | Lifestyle coaching, cooking activities, mindfulness, student goal-setting task. | Not specified. | Frequency not specified.  More than 80 hours total. | Not specified. |
| Bar Ilan University.  Israel. {47-48, 62-63 | Medicine.  1,2,3,4. | Nutrition, physical activity, stress reduction, reduction of avoidance of risky substances, sleep, sexual health, (behaviour change, motivational interviewing). | Public Health tutors. Physicians. | Elective or Mandatory.  Incorporated into main degree. | Lectures, exercise activity, yoga class, application of project in local community, small group discussions, MCQs, counselling, patient coaching, cooking activities, group discussions, mindfulness sessions, case-based learning and case presentations, role play with actors, reading food labels, food diary. | Not specified. | In 2015 - All hours in 1 day.  6 hours total.  In 2019 - 1 day at the beginning of 1st year and 2 days at the end of 1st year. 24 hours total.  In 2020 – Classes scattered throughout the 4 year medical degree. 58 hours total.  In 2022 - 6 sessions. 4 hours and 30 minutes total. | Likert scale survey of perceptions of programme, oral feedback on programme. Online questionnaire relating to self-perceived competence, health behaviours, attitudes and confidence. |
| Brandenburg Medical School.  Germany. [39] | Medicine.  All years. | Nutrition. | Dietician, chefs, previous students of programme, physician. | Elective.  Award not specified. | Interactive lecture, recipe discussion, cooking activities, tasting sessions. | Synchronous.  Online in 2021, in-person in 2022. | Once a week for 7 weeks or as block of 4-5 days.  28 hours total. | Self-assessment questionnaires on counselling knowledge, attitudes and competencies, Likert-scale habit assessment, WHO-5 wellbeing index MCQs, all pre and post-programme. |
| Brown University, Warren Alpert Medical School.  USA. [36] | Medicine. 3,4. | Nutrition, physical activity, stress management, sleep, social connection, avoidance of risky substances. | Not specified. | Elective.  Award not specified. | Clinical shadowing, movement therapy, yoga, mindfulness, cooking activities. | Not specified. | Daily for 2 weeks. Total hours not specified. | Not specified. |
| University of California Irvine School of Medicine.  USA. [36] | Medicine.  Pre-clinical years. | Nutrition | Chef, dietician, health coach. | Not specified. | Cooking activities, case studies, presentations. | Not specified. | Not specified. | Not specified. |
| University of Central Florida School of Medicine.  USA. [36, 44, 54, 70-72] | Medicine.  2,3,4. | Nutrition, physical activity, stress management, sleep, social connection, avoidance of risky substances, (Motivational interviewing). | LM boarded physician, psychologist, “Lifestyle Medicine expert”. | Elective & Mandatory programmes.  Credits. | Online lectures, Clinical shadowing, lifestyle histories, lifestyle prescriptions, providing nutrition education to patients, breathing exercises with patients, reading, case-based learning, reflections, student SMART goal-setting, group discussions, audience response activities, lifestyle assessments, MCQs, OSCE, problem solving clinical scenarios, peer to peer feedback, role-play | Hybrid (However, in 2019 online or in-person was offered, but not a hybrid option).  Hybrid. | In 2017 - 1 session.  2 hours total.  In 2019 - Classes spread throughout 12 week module. 1.5 hours total.  In 2020 - Once a week for 12 weeks. 3 hours total.  In 2023 - Daily for 2 or 4 weeks. Total hours not specified. | Attendance, assignment completion, oral feedback, faculty subjective satisfaction, grading of quality of student engagement, Kirkpatrick pyramid, Likert-scale questionnaire of student satisfaction, summative OSCE, scoring of SMART goals. Pre and post-programme Likert-scale questionnaire regarding confidence implementing LM in practice. |
| Edward Via College of Osteopathic Medicine Carolinas.  USA. [68] | Medicine.  2. | Nutrition, physical activity. | Physicians, chefs, dieticians. | Elective.  Award not specified. | Lectures, guest speakers, recipe creation, food tasting, cooking activities, volunteering at food initiatives. | Synchronicity not specified.  Hybrid. | 2 hours a month for 8 months.  16 hours total. | Pre and post-programme Likert questionnaire on confidence, competency and satisfaction. Monthly qualitative surveys of knowledge and confidence. |
| George Washington School of Medicine and Health Sciences.  USA. [49] | Medicine.  Year not specified. | Nutrition, physical activity. | Credits. | Not specified. | Patient history taking and lifestyle prescriptions. | Not specified. | Two 3 credit-hour programmes.  6 hours total. | Not specified. |
| University of Gieben.  Germany. [39] | Medicine.  All years. | Nutrition. | Dietician, chefs, previous students of programme, physician. | Elective.  Award not specified. | Interactive lecture, recipe discussion, cooking activities, tasting sessions. | Synchronous.  Online in 2021, in-person in 2022. | Once a week for 7 weeks or as block of 4-5 days.  28 hours total. | Self-assessment questionnaires on counselling knowledge, attitudes and competencies, Likert-scale habit assessment, WHO-5 wellbeing index MCQs, all pre and post-programme. |
| University of Gottingen.  Germany. [39] | Medicine.  All years. | Nutrition. | Dietician, chefs, previous students of programme, physician. | Elective.  Award not specified. | Interactive lecture, recipe discussion, cooking activities, tasting sessions. | Synchronous.  Online in 2021, in-person in 2022. | Once a week for 7 weeks or as block of 4-5 days.  28 hours total. | Self-assessment questionnaires on counselling knowledge, attitudes and competencies, Likert-scale habit assessment, WHO-5 wellbeing index MCQs, all pre and post-programme. |
| Hadassah-Hebrew University School of Medicine.  Israel. [42, 74] | Medicine.  1,2,3,4,5,6. | Nutrition, physical activity, avoidance of risky substances [as smoking cessation] (behavioural change). | GPs, sports physicians, exercise physiologists, health psychologists, public health physicians, social workers. | 3 mandatory modules, one elective module.  Award not specified. | Lectures, case studies and case presentations, history taking, webinars, online module, group discussions, experiential work, health coaching (friends in pre-clinical years, patients in clinical years), clinical shadowing and bedside teaching. | Hybrid.  Hybrid. | Classes distributed across 6 years.  58 hours total. | Frequency and content of student-student health-coaching interactions. Pre and post-programme questionnaires with Likert-scale questions assessing students self-perceived ability to deliver LM interventions, open-ended questions regarding student and faculty satisfaction with curriculum. |
| Harvard Medical School.  USA. [2, 50, 80] | Medicine.  1,2,3,4. | Nutrition, physical activity, (motivational interviewing). | Physician. | Elective.  No award. | Lectures, workshops. | Synchronous.  In-person. | One hour long lecture. | Pre and post-programme survey of knowledge, attitudes and confidence regarding lifestyle medicine, including multiple choice questions. |
| Imperial College School of Medicine in London.  UK. [51] | Medicine.  1,2. | Nutrition, physical activity, sleep, (mental health). | Clinicians. | Mandatory (incorporated into main degree).  15% of learning and assessment. | Flipped classroom, small group tutorials, reflections. | Not specified. | 15% of learning and assessment. | Questionnaire – Likert-scale and open-ended questions relating to importance of lifestyle medicine. |
| Loma Linda University, School of Medicine.  USA. [2, 36, 57] | Two programmes taught only medical students, another taught all students in the school of allied health professions. 1,2,3,4. | Nutrition, physical activity, stress management, sleep, social connection, avoidance of risky substances. | Occupational therapist, doctor. | Elective or LM track.  Entered into raffle for 50 dollar gift card. | Journal Club, clinical shadowing, cooking activities, wellness tracking, plant-based lunches, presentation, reading, personal improvement project, group presentations, nutrition tracking and prescription, 5 minute video, list of resources, interactive activities, student goal-setting, diet reflection, motivation and reward identification, exercise prescription, mindfulness practice, journalling. | Asynchronous.  Online. | The medical student elective was daily for 2 weeks. 50 hours total.  The LM track contained over 80 hours of dedicated LM teaching.  The 2024 programme for all students was 5 minutes per week for 4 weeks.  20 minutes total. | Participants completed the Depression, Anxiety, and Stress Scale (DASS-21) and the Health-Promoting Lifestyle Profile II (HPLP-II) before and after the program. Additionally, they completed a 7-item post-program survey and engaged in semi-structured interviews approximately one month post participation. |
| Mayo Clinic Alix School of Medicine in Arizona.  USA. [43] | Medicine.  1. | Nutrition, Physical activity, stress management, sleep. | Multidisciplin-ary clinicians. | Mandatory. Award not specified. | Flipped classroom (online module + discussions), videos, lifestyle self-assessment, client coaching, didactics, small group discussions, label reading, virtual grocery store tour, diet prescription, meal plan with budget, documentaries with accompanying discussions, food tasting, fitness tests, exercise prescriptions, stress self-assessment, mindfulness practice. | Hybrid.  Hybrid. | Programme spread over 3 days.  11 hours total. | Pre and post-programme Likert-scale survey assessing knowledge of and attitude towards topics. Open-ended qualitative questions. |
| University of North Carolina.  USA. [75] | Medicine.  3,4. | Nutrition, sexual health. | Not specified. | Elective.  Award not specified. | Lectures, small group discussions, cooking activities, online modules. | Hybrid.  Online. | Not specified. | Pre and post-programme surveys regarding confidence implementing LM in practice. |
| Northeastern University Bouvé College of Health Sciences.  USA. [79] | Physician associates.  Year not specified. | Nutrition, physical activity, sleep, stress management, avoidance of risky substances, social connection. | Not specified. | Mandatory.  Award not specified. | Lectures, videos, meditation, breathing exercises, lifestyle prescriptions. | Not specified. | 8 lectures.  Hours not specified. | Not specified. |
| Northwestern University, Feinberg School of Medicine.  USA. [45, 52, 58, 59] | Medicine.  1,2,3,4. | Nutrition, physical activity, avoidance of risky substances sleep, stress (behaviour change, body weight). | Not specified. | Mandatory.  Incorporated into main degree as one of 5 curricular threads. | Coaching elementary students in nutrition, an external culinary medicine program, personal behaviour change plan, self-reflection, written examination, MCQs, scholarly project, history taking, lectures, prescribing, OSCEs, small group assessments, cardiopulmonary fitness measurements. | Not specified. | 81 sessions spread over 2 years. 81 hours total. | Traditional measures of knowledge and clinical performance. Longitudinal portfolio. |
| Ohio University.  USA. [67] | Medicine.  Year not specified. | Nutrition. | Not specified. | Not specified. | Video, articles, lectures, clinical shadowing. | Not specified. | Frequency not specified.  2 hours total. | Not specified. |
| University of Oklahoma.  USA. [2, 80, 84] | Medicine.  1,2. | Nutrition, sleep, physical activity, stress management. | Not specified. | Elective and mandatory elements.  Award not specified. | Didactic, experiential, meditation, mindfulness, cooking activities including culinary medicine, community outreach, simulated patients, research, community class facilitation. | Not specified. | Frequency not specified.  69 hours total. | Not specified. |
| Quinnipiac university.  USA. [61, 77] | Medicine.  Pre-clinical years. | Nutrition, physical activity, sleep, stress management, avoidance of risky substances, social connection. | Physicians, faculty & alumni. | Elective and mandatory programmes.  Award not specified. | Didactics, case studies, workshops, guest speakers, book clubs, reflections, receiving coaching from programme alumni, drawing a dinner plate exercise, group discussions, deep breathing activity, walk, presentation. | Synchronous.  In-person. | In 2020 - 3 workshops of 1 hour each.  3 hours total.  In 2023 – 2-3 hour-long sessions over 16 weeks.  2-3 hours. | Pre and post-programme Likert survey regarding self-perceived knowledge, skills and attitudes towards LM. |
| Riphah international university.  Pakistan. [78] | Medicine.  1,2,3. | Nutrition, physical activity, sleep, stress management, avoidance of risky substances, social connections (behaviour change, positive psychology, empowering self and others). | Not specified. | Mandatory.  Award not specified. | Lectures, problem based learning, skill labs, history taking, clinical rotations, case studies, MCQs, OSCEs, VIVA, research projects. | Not specified. | Not specified. | Not specified. |
| University of Rochester, School of Medicine and Dentistry.  USA. [69] | Medicine.  3,4. | Nutrition, stress management, physical activity, (Informally: sleep, social connection and substance use) | LM practitioners. | Elective.  Award not specified. | Clinical observation, reading, patient coaching, plant diet challenge, student SMART goal setting task, meditation, walking meetings. | Synchronicity not specified.  Hybrid. | 2 week block of classes.  Total hours not specified. | Post-programme questionnaire on understanding of LM and confidence implementing LM in practice. Evaluation of knowledge and skills by preceptors. |
| University of Southern California (USC), Keck School of Medicine (KSOM).  USA. [46] | Medicine.  1. | Nutrition, physical activity. | Not specified. | Mandatory within primary care track.  Award not specified. | Lecture, videos, reading, teaching exercise classes to local communities. | Synchronicity not specified. Hybrid. | 1 hour lecture & 35 minutes teaching exercise classes to patients. | Post-survey with closed and open-ended questions relating to perceived knowledge, confidence and satisfaction. |
| University of South Carolina.  USA. [2, 38, 67, 80, 82, 41] | Medicine. 1,2,3,4. | Nutrition, Physical activity, sleep, stress management, (behaviour change). | Dieticians, exercise physiologists, wellness coaches, chefs. | Mandatory & electives.  Award not specified. | Lectures, case studies, OSCEs, reflections, cooking activities, nutrition reports, plate presentations, patient interactions, problem based learning, simulated patients, role play, clinical experiences, exercise prescriptions, dietary counselling, behavioural change counselling (motivational interviewing), planting crops, bike rides. | Not specified. | Classes distributed throughout 4 years.  86.5 hours total. | Summative exam performance statistics. Student evaluations of faculty. |
| Stony brook university.  USA. [80] | Medicine.  Year not specified. | Nutrition, physical activity, sleep, stress management. | Family physician, dietician, social worker, psychiatrist. | Both.  Award not specified. | Lectures, literature reviews, discussion groups, cooking activities, presentations, guideline reviews, clinical observations, role-playing, field trips. | Not specified. | Not specified. | Not specified. |
| Texas College of Osteopathic Medicine.  USA. [60] | Medicine.  Year not specified. | Nutrition. | Not specified. | Nutrition elective.  Award not specified. | Not specified. | Not specified. | Not specified. | Survey relating to student perceptions of LM and health behaviours. |
| University of Texas Southwestern Medical School.  USA. [60] | Medicine.  1,2,3,4. | Nutrition (others informally). | Not specified. | Nutrition elective, LM informally integrated into main degree.  Award not specified. | Lectures. | Taught within other modules. | Not specified. | Survey relating to student perceptions of LM and health behaviours. |
| University of Toledo.  USA. [40, 65, 66, 67] | Medicine.  1,2. | Nutrition, physical activity, stress management, avoidance of risky substances (as smoking cessation). | Dietician, faculty physicians. | Elective.  Course credit. | Lectures, student SMART goal-setting task, mindfulness, cognitive restructuring, breathing exercises, mindful eating, case studies, treatment plans, personal lifestyle assessment, group discussions, case studies. | Synchronous.  In-person, though online in 2020. | In 2019 - 7 sessions over one semester.  5 hrs 35 minutes total.  In 2020 – not specified.  In 2021 - 5 minutes per week for 4 weeks.  20 minutes total. | Pre and post screening tests: GAD-7, fruit and vegetable screening assessment, perceived stress scale, PHQ9, minutes of physical activity, steps, dietary fat. Likert scale rating usefulness of course and progress towards lifestyle goal achievement. |
| Tulane University School of Medicine.  USA. [38] | Medicine.  Year not specified. | Nutrition, Physical activity, sleep, (behaviour change). | Not specified. | Not specified. | Lectures, hands-on culinary medicine experiences. | Not specified. | Not specified. | Not specified. |
| University of Tulsa.  USA. [84] | Physician associate.  2. | Nutrition, physical activity, avoidance of risky substances (body weight reduction). | Physician, dietician, chef. | Elective/mandatory nature not specified.  2 credits. | Didactics, cooking activities, case studies, quizzes, reflections, online modules, group project. | Hybrid.  Hybrid. | 4 weeks.  2 credit hours. | Pre and post-programme survey,of student perceptions of knowledge and confidence, qualitative open-ended questions relating to experience of programme, MedDiet score, BMI. |
| Virginia tech Carilion school of medicine.  USA. [67] | Medicine.  3. | Nutrition, physical activity, stress management, avoidance of risky substances, sleep, social connection. | Physician, public health educator, exercise physiologist. | Not specified. | Lectures, experiential learning, behaviour diary, case studies. | Not specified. | Frequency not specified.  3 hours total. | Measurements of student confidence in LM prescriptions. |
| Wake Forest School of Medicine.  USA. [55] | Medicine.  1. | Nutrition, physical activity, stress management, sleep. | Student-led. | Elective.  Award not specified. | Building a balanced plate exercise, muscle and bone building exercises, grocery shopping challenges, budget kitchen building exercise, food diaries, sleep diaries. | Not specified. | Contained within programme.  Total hours not specified. | Not formally evaluated. |
| Western New England University.  USA. [64] | Pharmacy.  3. | Nutrition, physical activity, sleep, stress management, avoidance of risky substances, (motivational interviewing). | Not specified. | Elective.  3 credits. | Student goal-setting and completing, reflections, relaxation videos, muscle relaxation exercises, yoga, taste-testing organic vs non-organic foods, colour therapy, meditation, financial wellness planning, stretching, pet therapy, mindfulness, lectures, quizzes, food diaries, patient cases, role-playing, designing patient care plans, composite lifestyle index tool, fitness testing, group presentations. | Not specified. | Twice a week for 15 weeks.  60 hours total. | Pre and post-programme survey on perceptions of LM and confidence in implementing LM interventions, pre and post-programme fitness test, examinations assessing concept comprehension. |
| Western University of Health Sciences College of Osteopathic Medicine of the Pacific.  USA. [67] | Medicine.  All years. | Nutrition, physical activity, stress management, avoidance of risky substances [as smoking cessation], (weight loss, self-care, mindfulness). | Not specified. | Mandatory & elective components.  Award not specified. | Community service. | Not specified. | Frequency not specified.  8 hours total. | Not specified. |
| West-Virginia School of Osteopathic Medicine.  USA. [34] | Medicine  3,4. | Nutrition, physical activity, stress management. | Clinical exercise physiologist, diabetes care specialist, chef. | Elective. Award not specified. | Video lectures, handouts, reading, quizzes, lectures, literature reviews, online modules, diabetes counselling, group discussions, exercise prescription, fitness tests (VO2 max, body composition, muscular strength/endurance, flexibility) , exercise demonstrations, field trip (shopping for groceries), yoga, mindfulness, cooking lab, clinical shadowing, group discussions. | Hybrid. Hybrid. | Classes all day, daily, for 2 weeks. 68 hours total. | Pre and post-programme Likert-scale survey of perceptions of topic and experience of programme, open-ended qualitative questions. |
| West Virginia University School of Medicine.  USA. [53] | Medicine.  1,2,3,4. | Nutrition, physical activity, stress management, sleep | Psychiatrist, paediatrician, cardiologist, internal medicine physician, endocrinologist, exercise physiologist, behavioural medicine psychologist, chef, culinary medicine certified family medicine physician. | Optional degree track.  Track qualification and optional culinary medicine certificate. | Lectures, readings, quizzes, cooking activities, journal clubs, clinical learning groups, summer externships, clinical electives, capstone project, elective culinary medicine certification. | Synchronicity not specified.  Hybrid. | Frequency not specified. 300 hours total over 4 years. | Qualitative and quantitative pre and post-programme surveys relating to perceptions of attendance, projects and the Certified Culinary Medicine Specialists exam. Number of students completing the Certified Culinary Medicine Specialists exam, number of students applying to track, number of students completing the final project. |
| **Programmes that taught pre-registration healthcare students but either did not specify the university or were run by an organisation that is not a university.** | | | | | | | | |
| **Organisation, or author if no organisation was specified.** | **Degree being taught & Year of degree** | **Pillars of LM**  (Core topics named but outside conventional 8 pillars) | **Tutors profession** | **Nature of programme & Award** | **Teaching & assessment methodologies employed.** | **Synchronicity & Mode of content delivery** | **Frequency of classes & Hours of tutelage** | **Method of programme evaluation & aspect being assessed.** |
| Balasubramaniam et al.  2021. (Author) [35] | Not specified. | Not specified. | Not specified. | Elective.  Award not specified. | Students coached LM to children. | Not specified. | Not specified. | Qualitative feedback & Likert scale surveys of confidence in knowledge, enjoyment of programme and relevance of content. |
| Basha et al.  2021. (Author) [37] | Nursing.  Year not specified. | Nutrition, physical activity, sleep, stress management, avoidance of risky substances. | Not specified. | Not specified. | Student lifestyle modification goal, tracking & reflection. | Not specified. | 2 week-long assignment.  Total hours not specified. | Survey of student learning perceptions. |
| ‘Health meets food curriculum’ – many unspecified universities in the USA. [76] | Medicine.  Year not specified. | Nutrition. | Physicians, dieticians, chefs. | Mandatory in some universities, elective in others.  Award not specified. | Nutrition counselling, cased based learning, cooking activities, didactics, discussions | Synchronous.  Either online or in-person (not hybrid). | One 4 hour session per week for 8 weeks. 32 hours total. | Pre and post-programme survey with Likert-scale questions relating to student attitudes, habits and self-perceived counselling competencies. MedDiet score. |
| Keyes & Gardner.  2020. (Author) [56] | Physician associates.  1. | Nutrition, physical activity, stress management, avoidance of risky substances (as smoking cessation). | LM certified faculty member. | Elective.  Award not specified. | Lifestyle history and lifestyle prescription, guideline learning. | Not specified. | 4 classes of 50 minutes.  3 hours and 20 minutes total. | Survey of self-perceived competency pre and post programme. Prevention and lifestyle assessment write-up. Formally assessed critical thinking session. |
| VA Boston healthcare system. [73, 81] | Physician associates.  1,2,3. | Nutrition, physical activity, stress reduction, (motivational interviewing). | Clinicians, physiotherapists, certified health coach. | Mandatory.  None. | Didactics, clinical observation, personal self-care activities, exercise prescriptions, cooking activities, presentation, reflection. | Hybrid.  Hybrid. | Classes distributed across 5 weeks. 12 hours. | Multiple choice quiz, self-efficacy questionnaire. Clinical vignettes pre and post programme. |
| Visaria et al.  2022. (Author) [83] | Not specified. | Not specified. | Not specified. | Not specified. | Online module, simulated patient assessment, supervised community screening, blood pressure measurements, lifestyle history, lifestyle counselling. | Synchronicity not specified.  Hybrid. | Not specified. | Content-based MCQ pre and post-training. |
